# Silver Can Induce Oxidative Stress in Parallel to Other Chemical Elicitors to Modulate the Ripening of Chili Cultivars

**DOI:** 10.3390/plants9020238

**Published:** 2020-02-12

**Authors:** Arijit Ghosh, Indraneel Saha, Debabrata Dolui, Arnab Kumar De, Bipul Sarkar, Malay Kumar Adak

**Affiliations:** Plant Physiology and Plant Molecular Biology Research Unit, Department of Botany, University of Kalyani, Kalyani, Nadia-741235, India; arijitgo625@gmail.com (A.G.); indraneelsaha92@gmail.com (I.S.); debabratabotany@gmail.com (D.D.); akde26@gmail.com (A.K.D.); bipulsarkar007@gmail.com (B.S.)

**Keywords:** oxidative stress, ripening physiology, silver, chemical elicitors, chili

## Abstract

Two chili cultivars, i.e., cv. Bullet and cv. Tejaswini, were evaluated on postharvest related ripening characteristics with varying durations under hydrogen peroxide, putrescine and silver treatments. The reducing sugar was inversely related to the maximum values at 7 days of ripening. Silver and putrescine were the most regulatory in terms of changing of the total carbohydrate content as compared to hydrolysis of the total reducing sugar. Regarding pectin methylesterase activity, both chilies were consistent, regardless of the number of days of incubation. Still, putrescine and silver were significant contributors to variations in cv. Bullet and cv. Tejaswani. For the pigment content, lycopene and chlorophyll increased in a linear manner, although these treatments significantly varied over time. Hydrogen peroxide and putrescine were responsible for the maximum accumulation of lycopene for both the cultivars, whereas, only cv. Tejaswani displayed maximum carotenoid for putrescine. Silver for both chili varieties was the most inhibitory for lycopene and carotenoid content. Superoxide had a good impact on the accumulation of lipid peroxides, irrespective of the chili variety. The maximum accumulation of lipid peroxide was recorded at seven days of treatment. Phenolics and flavonoids were in decreasing order for both the chili varieties, progressing through the days of the study period in a similar manner. Silver was the main contributor to variations in the phenolics and flavonoid contents in cv. Tejaswani. The solubilization of total carbohydrate into reducing sugar was in an inverse relationship, with the maximum values being reached at 7 days of ripening.

## 1. Introduction

Fruit ripening is established as a collective physiological process, with turnover as well the synthesis of bio-molecules being major events during fruit maturation. Changes of color, texture, and the solubilization of stored carbohydrates and other complex bio-molecules are facilitated by some gene expressions. This is more attributed to the elevation of CO_2_ concentration through increased respiratory rates [1]; however, this is the case mostly for climacteric fruits. For non-climacteric ones, it is not essentially accompanied with any changes in CO_2_ efflux. From a biochemical point of view, the total soluble solids and their conversion into sugars, the conversion of organic acids to keto-sugars, the turnover of flavon compounds are the most important. Leto-sugars, dextrans, and other disaccharides are responsible for the major variations in the characteristics of fruit types [2]. Fruit ripening and abscission are facilitated by programmed cell death, and are regulated by some signaling mechanisms [3]. Both endogenous compounds, like growth substances and secondary metabolites, are also involved in the ripening processes. Likewise, abiotic factors including the accumulation of minerals in soil, temperature fluctuations, UV intensity, and hypoxic or anoxic exposure, can cause variations in the contents of flavonoids and phenolic compounds. Incidentally, all these physical and chemical elicitations are targeted on a cellular level, with a characteristic change in the redox of ripening tissues. Consequently, ripening fruit are highly exposed to a burst of ROS depending upon the fruit type [4]. The exogenous application of chemical residues as elicitors has been observed to modulate fruit ripening with a precise ratio of ROS generation, as well its degradation. Moreover, a precise ratio of the concentration and function on different biochemical reactions is also proportionate to ROS metabolism [5]. In postharvest physiology, the signaling through some elicitors, which may be either endogenous or even exogenously applied synthetic moieties, is quite relevant. Besides silver, other chemical moieties have been observed to modulate the ripening characteristics, including fruit texture, color, softness etc. On a cellular level, changing the bonding pattern of cell wall residues is related to an extension of the shelf life of fruits. A certain level of ROS accumulation with wall bound NOX activity may represent a characteristic biomarker for fruit ripening phenomena. The maturation of fruit varieties through changes in specific characteristics is also regarded as being related to biomarkers under conditions of chemical elicitation, with silver being the main contributor. This biomarker would be more aligned to the ROS metabolism of ripening fruit tissues, as well as ROS related gene expression [6]. Therefore, the analysis and correlation of those cellular responses as biomarkers would comprise unraveling the key factors in the ripening processes. This may be worthy of closer examination with a suitable fruit sample. *Capsicum* has been under investigation for both its qualitative and quantitative nutritional traits due to the acquisition of secondary metabolites [7]. *Capsicum*, a non-climacteric fruit, has also been important as a nutritional supplement with antioxidation properties. This could be important in horticultural practices, with induced or enhanced changes in ripening patterns during the postharvest period. This study sets out to monitor oxidative exposure and its impact on the ripening process in two distinctly different *Capsicum* varieties. Additionally, it seeks to determine the efficacy of a few chemical elicitors, including silver, during postharvest storage.

## 2. Results

In our observations, distinguished phenotypic changes were recorded as plants responded to different treatments. Besides this, the cellular and physiological responses of two chili varieties (Bullet and Tejaswani) as a function of three elicitors and their interactions during ongoing postharvest storage were validated statistically by Analysis of Variance (ANOVA) following determination of the Least Significant Difference (LSD) (Tables S1–S10).

### 2.1. Effect of Chemical Treatments on Lycopene and Carotenoid Content

**Figure 1:**
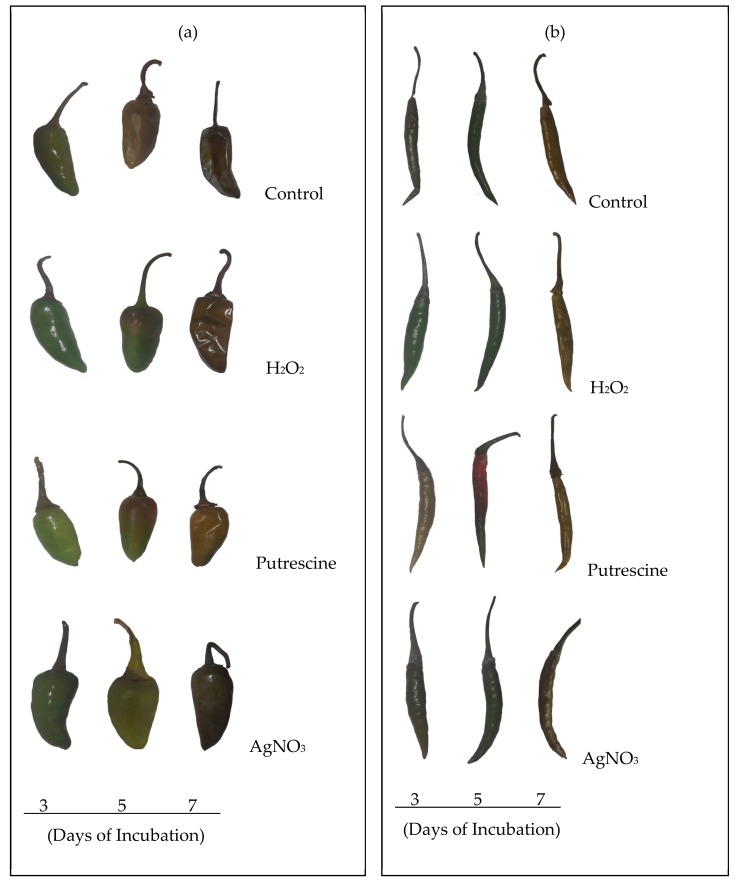
Morphological changes of chili fruits: *cv*. Bullet (**a**) and *cv*. Tejaswani (**b**) under various treatments, i.e., H_2_O_2_, putrescine, and AgNO_3_ after 3, 5, and 7 days.

**Figure 2:**
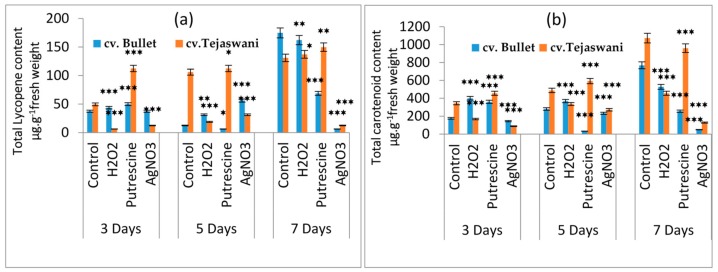
Changes of total lycopene (**a**) and total carotenoid content (**b**) of two chili types, i.e., *cv.* Bullet and *cv*. Tejaswani, under different treatments (Control, H_2_O_2_, putrescine and AgNO_3_) and durations (3, 5, and 7 days). The vertical bars represent the data with the mean of the three replicates with *± SE* (*n* = 3) from an individual set of experiments, and significant differences between treatments, as calculated by a student’s t-test marked as * (*p* ≤ 0.05) ** (*p* ≤ 0.01) *** (*p* ≤ 0.001).

On the basis of chemical treatments, the two varieties of *Capsicum* fruit varied in their responses in a distinct manner. Monitoring of the delayed changes related to ripening was undertaken with consideration of varietal differences. In the initial observation, a detachment or loosening of the fruit stalk was observed, which is an indication of senescence. However, this varied significantly with duration during the treatments, compared to the control. As compared to the control, regardless of the chemical treatments, changes in fruit color were not prominent, even up to 3 days, but became distinct after 5 and 7 days (Figure 1). At 7 days, the contents of pigments such as lycopene and carotenoids in the fruit were significantly varied compared to the control throughout the treatments. As the fruit progressed through the days of incubation, the lycopene content was maximally affected at 7 days, regardless of the variety (Figure 2a). However, significant variation was observed with treatment with putrescine, but not to the same extent for *cv*. Bullet and *cv*. Tejaswani, with a 60.74% decrease and a 0.14-fold increase, respectively compared to the control. Still, in both chili varieties, AgNO_3_ also showed significant effects by the down regulation of lycopene content throughout the days of treatment compared to the control. The maximum levels of regulation were 90.48% and 96.42% fall for *cv*. Tejaswani and *cv*. Bullet compared to the control at 7 days. For carotenoids, the activities for both chili varieties were also significantly varied (*p* < 0.01, 0.05) under each treatment (Figure 2b). Thus, the AgNO_3_ appeared to be down regulatory for carotenoid, by 88.02% and 93.75% compared to the control. In contrast, putrescine treatment was discriminatory for carotenoid content for the two chili variety. At 7 days, putrescine significantly (*p* ≤ 0.001) reduced the pigment content by 69.01% for *cv*. Bullet, whereas for *cv*. Tejaswani, it was only 10.45%.

### 2.2. Changes of Reducing Sugar and Total Sugar Content

**Figure 3:**
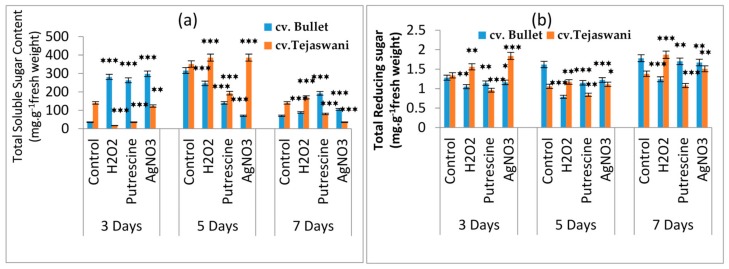
Changes of total soluble sugar (**a**) and total reducing sugars (**b**) of two chili types, i.e., *cv.* Bullet and *cv*. Tejaswani, under different treatments (Control, H_2_O_2_, putrescine, and AgNO_3_) and durations (3, 5, and 7 days). The vertical bars represent the data with means of three replicates with *± SE* (*n* = 3) from an individual set of experiments, and significant differences between treatments, as calculated by a student’s t-test marked as *(*p* ≤ 0.05) **(*p* ≤ 0.01) ***(*p* ≤ 0.001).

The most significant turnover of metabolites in the fruit ripening process is from sugar profiles. Significant changes in the total sugar and reducing sugar contents of the two chili varieties are presented in Figure 3a,b respectively. In both varieties, over the course of the treatment period, reducing sugar and total sugar contents with all treatments were quite discriminatory with respect to control. Compared to the control at 7 days, the reducing sugar contents in all the treatments were maximally changed for both chili varieties. Treatment with H_2_O_2_ was shown to change the sugar content, yielding a 0.35-fold increase for *cv*. Tejaswani, but a 30.34% decrease in the case of *cv*. Bullet chilies at 7 days. Similar trends were also observed on days 3 and 5 under H_2_O_2_ treatment. For *cv*. Tejaswani, the changes of total soluble sugar content were almost the opposite, showing a 21.74% decrease and 0.09-fold increase under putrescine and AgNO_3_ at 7 days, compared to the bullet chili variety. This change in total sugar content was brought forward from days 3 and 5, but was not necessarily always significant (*p* ≤ 0.01, 0.001). Two varieties were significantly distinct in their trend of total soluble sugar accumulation at 5 and 7 days. This was attributed to the H_2_O_2_, putrescine, and AgNO_3_ treatments for both varieties. At 5 days, with the exception of the putrescine treatment, an increase of total carbohydrate was recorded in *cv*. Tejaswani: 0.09-fold and 0.09-fold increases respectively for H_2_O_2_ and AgNO_3_. *cv*. Bullet chilies, in contrast, showed a reduced carbohydrate content, i.e., 22.15%, 55.69%, and 77.85% for the H_2_O_2_, putrescine and AgNO_3_ treatments, compared to the control. On the 7th day, the Bullet chili variety accumulated sugar maximally under H_2_O_2_, putrescine, and AgNO_3_. For the tejaswani variety, a significant promotional effect, i.e., a 0.21-fold increase was recorded only with H_2_O_2_ treatment at 7 days. However, in the Tejaswani variety, the putrescine and AgNO_3_ treatments were quite inhibitory, i.e., 42.86% and 75% reductions of total sugar contents at 7 days.

### 2.3. Influence of Treatments on H_2_O_2_ and Lipid Peroxide Production

**Figure 4:**
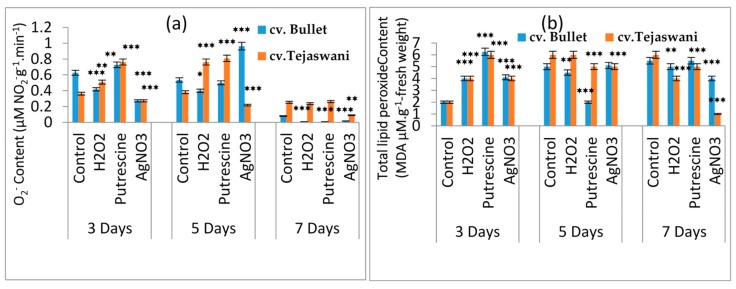
Changes of total O_2_^-^content (**a**)and total lipid peroxide content (**b**) of two chili types, i.e., *cv.* Bullet and *cv*. Tejaswani, under different treatments (control, H_2_O_2_, putrescine, and AgNO_3_) and durations (3, 5, and 7 days). The vertical bars represent the data with means of three replicates with ± SE (*n* = 3) from the individual set of experiments, and significant differences between treatments as calculated by student’s t-test marked as *(*p* ≤ 0.05) ** (*p* ≤ 0.01) *** (*p* ≤ 0.001).

The ripening event through physiological intervention was also facilitated by the induction of reactive oxygen species (ROS). This idea also materialized in the present experiment through the accumulation of O_2_^-^ in both chili varieties during incubation under all treatments for 3, 5, and 7 days. Both the varieties were maximally affected at 7 days with significant (*p* ≤ 0.01, 0.001) decreases in O_2_^-^ contents compared to days 3 and 5 with respect to the control (Figure 4a). Therefore, the most striking effects were the gradual loss of O_2_^-^ by 60.86%, 60.86%, and 30.43% for *cv*. Bullet under H_2_O_2_, putrescine, and AgNO_3_ respectively. In contrast, the Tejaswani chili variety could not modulate its O_2_^-^ content under H_2_O_2_ and putrescine, but reduced it significantly under AgNO_3_, i.e., by 42.86% at 7 days compared to the control. In contrast, at 5 days, it was more promising and consistent in terms of the up regulation of O_2_^-^for all treatments on the Tejaswani chili variety. As a consequence of O_2_^-^ generation, the changes of membrane permeability were also quite expected, with the generation of lipid peroxide content in both varieties. The trend in MDA concentration throughout treatments showed an inconsistency with a rise and fall which was observed in both chili varieties (Figure 4b). As in the example, at 3 days, the Bullet variety became more prone to oxidative damage by increased MDA content compared to the Tejaswani variety. In contrast, on day 5, the *cv*. Tejaswani was shown to produce a higher MDA content then *cv*. Bullet, irrespective of treatments. However, the maximum variability was observed at 7 days, where the H_2_O_2_ treatment yielded a 6.78% and 32.31% decrease in the MDA content compared to the control for the Bullet and Tejaswani varieties, respectively. In the Tejasani variety, putrescine was less effective under the same conditions, whereas silver yielded a reduced MDA content, with a significant (*p* ≤ 0.001) variation. Thus, *cv*. Bullet and *cv*. Tejaswani showed reduced levels of MDA by 22.03% and 83.08% as compared to the control.

### 2.4. Changes of Phenolic Content and total Flavonoid Content

**Figure 5:**
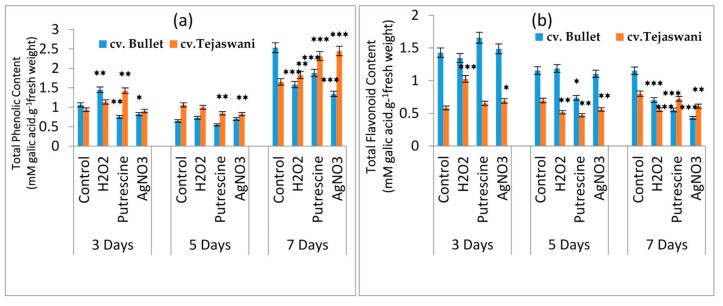
Changes of total phenolic content(**a**)and total flavonoid content (**b**) of two chili types, i.e., *cv.* Bullet and *cv*. Tejaswani, under different treatments (Control, H_2_O_2_, putrescine, and AgNO_3_) and durations (3, 5, and 7 days). The vertical bars represent the data with the means of three replicates with ± SE (*n* = 3) from an individual set of experiments, and significant differences between treatments as calculated by a student’s t-test marked as *(*p* ≤ 0.05) **(*p* ≤ 0.01) ***(*p* ≤ 0.001).

Important constituents for fruit ripening are the phenolic residues and their corresponding derivatives. A turnover of phenolic content was observed in *cv*. Bullet and *cv*. Tejaswani throughout the experiment period (Figure 5a); the observed maximum at 7 days indicated the involvement of phenolic residues up to the final stages of ripening. The maximum changes at 7 days were recorded in all the treatments for both varieties with up and down regulation. Thus, the *cv*. Tejaswani variety had a linear increase of phenol content compared to the control, i.e., increases by 0.1 fold, 0.4 fold, and 0.48 fold under H_2_O_2_, putrescine, and AgNO_3_ treatments, respectively. In contrast, the Bullet chili variety was also sensitive those treatments, showing 37.48%, 25.65%, and 46.85% increases under H_2_O_2_, putrescine, and AgNO_3_ treatments, respectively. Flavonoids and other important residues for total phenolic content were more involved in the initial days of the treatments. A gradual decline in flavonoid content undoubtedly indicated depletion through the fruit ripening process (Figure 5b). In the present experiment, the Tejaswani variety was inconsistent in the up and down regulation of flavonoid content. At 3 days, however, H_2_O_2_ was shown to have up regulated the content by 0.76 fold compared to the control. *cv*. Bullet did not show any significant variation with the treatments; however, the peak in the flavonoid content recorded with putrescine increased by 0.138 fold at 3 days.

### 2.5. Activity of Pectin Methylesterase (PME) under Different Treatments

**Figure 6:**
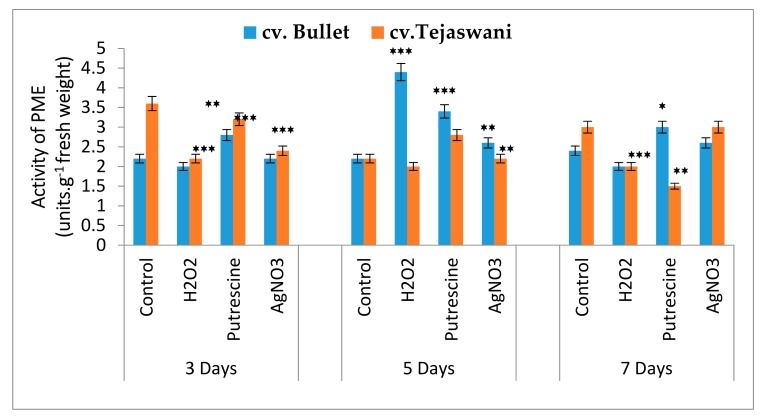
Assay of PME activity of two chili types, *cv.* Bullet and *cv*. Tejaswani, under different treatments (Control, H_2_O_2_, putrescine, and AgNO_3_) and durations (3, 5, and 7 days). The vertical bars represent the data with the mean of three replicates with ± SE (*n* = 3) from an individual set of experiments, and significant differences between the treatments as calculated by a student’s t-test marked as *(*p* ≤ 0.05) **(*p* ≤ 0.01) ***(*p* ≤ 0.001).

PME is a cell wall lysing enzyme that was shown to be the most discriminatory (Figure 6). However, chemical treatments were varied, with differential patterns of enzyme activities at 3,5, and 7 days for both varieties. An up regulation of PME activity compared to the control was maximized for the Bullet variety, then the Tejaswani variety at 3 days under all the treatments. This trend reversed for the *cv*. Bullet, with maximum activity through H_2_O_2_, putrescine, and AgNO_3_ at 5 days. At 7 days, an admixture of heterogeneity in terms of the activity between *cv*. Bullet and *cv*. Tejaswani was recorded. Thus, H_2_O_2_ did not yield any significant changes for the two chili varieties, but for putrescine and AgNO_3_, the activities were distinct between two varieties. With putrescine, the activities were 50% subdued for *cv*. Tejaswani, which increased by 0.25 fold for *cv*. Bullet compared to the control. On the other hand, AgNO_3_ remained non-significant for both chili varieties against the control at 7 days.

### 2.6. Bioaccumulation of Silver in the Tissue

**Figure 7:**
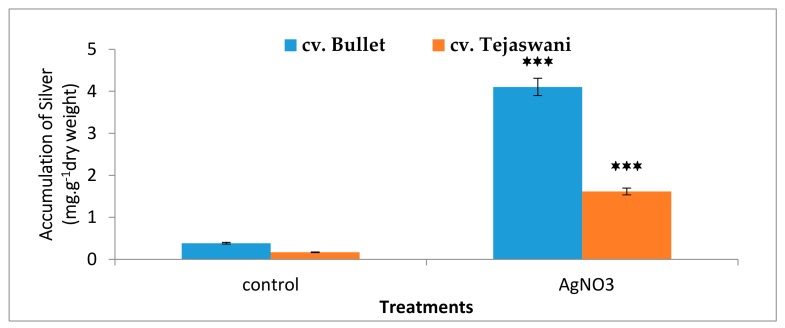
Changes in accumulation of silver of two chilitypes, *cv.* Bullet and *cv*. Tejaswani, under treatments (control and AgNO_3_) after 7 days. The vertical bars represent the data with the mean of three replicates with *± SE* (*n* = 3) from an individual set of experiments, and significant differences between the treatments as calculated by a student’s t-test marked as *(*p* ≤ 0.05) **(*p* ≤ 0.01) ***(*p* ≤ 0.001).

As expected from the silver nitrate treatment, the chili varieties were compatible in metal accumulation. Figure 7 illustrates the accumulation of silver in the pulp, albeit one that varied in concentration in the two varieties at 7 days. As compared to the control, a significant (*p* ≤0.05) accumulation was recorded, i.e., 10.71 and 9.67 fold respectively, in cv. Bullet and cv. Tejaswani. Therefore, the tissue flexibility is up taken with metals. The responses of silver as an inducer are likely to be variable for the two chili varieties.

## 3. Discussion

The ripening of fruit is a developmental process where physiological cellular events occur in plants. This research examined the delayed ripening of fruits in postharvest storage. It also proposes a physical/chemical system for the prolonged storage of fruits. In the present experiment, a chemical system employing H_2_O_2,_ putrescine, and silver was used to extend the maximum shelf life of chili fruits under ambient laboratory conditions. From the aforementioned results, the efficacy of these chemicals is discussed in relation to the modulation of the most significantly contributing elements over the course of the treatments. The pigment system, including lycopene and carotenoids, the conversion of reserve carbohydrates into simpler forms, the acquisition of ROS and its impacts, the accumulation of secondary metabolites, the fruit skin’s softening enzymes, and finally, the absorption of metals like silver into fruit tissue, were important. These contributed to the overall ripening phenomenon, either directly and indirectly [8]. Silver, a toxic metal, remains effective in threshold concentrations in terms of influencing ripening [9]. Of the chemical elicitors used in earlier studies, silver has been shown to be able to induce some degenerative effects following concomitant disorders on human health, albeit beyond threshold concentrations in tissues [10]. In contrast, this metal has a unique ability, i.e., controlling ethylene functionales by blocking one of their receptors. Silver has been in demand, regardless of fruit type (climacteric or non-climacteric) in postharvest storage [11]. Therefore, the question of whether and how such a metal in minimal concentrations might be a modulator along with H_2_O_2_ and putrescine is worthy of exploration. The present study is a description in which two chili varieties were evaluated in terms of their cellular and physiological responses when interacting with silver ion-mediated ROS. This was elucidated with two chemical elicitors i.e., H_2_O_2_ and putrescine, which had modulated responses in their own way [12,13]. The discriminatory responses of fruits of those cultivars under the influence of the aforementioned chemical elicitors made it more complex to decipher the ripening process throughout the postharvest period. Likewise, the readily observable characteristic of fruit color, as measured herein, regarding lycopene and carotenoid contents, were interesting to study. The regulation of pigment content in the fruit throughout the ripening process indicates the metabolic status of tissues due to other interfering reactions. Thus, throughout the various stages of ripening (at 3, 5, and 7 days), an almost consistent trend of pigment content (lycopene and carotenoid) was recorded. The retaining of lycopene, a carotenoid, is important for two basic processes: quenching of indigenously produced ROS, and as a ripening index on the fruit’s skin [14]. Plant responses to oxidative stress vary according to genotypic specificity. Thus, increases in the total soluble sugar content at 5 days for the Tejaswani chili variety might be due to some changes in carbohydrate metabolism after oxidative stress. ROS often induces some sort of anomaly in tissue hydration/turgidity vis-à-vis water deficit [15]. It would be logical to examine the increase in sugar content linked to osmotic adjustment under such an ROS-induced water deficit. Both chili varieties might overcome the effect of H_2_O_2_ and thereby sustain the fruit skin integrity, leading to delayed ripening. This is also true for both chili varieties but may not be the case with AgNO_3_. Silver has an intrinsic ability to modulate the ripening process, either by the down regulation of ethylene biosynthesis or by other, related phenomenon [16]. Thus, for both varieties, silver had down regulated the lycopene and carotenoid contents. In the context to polyamine, (putrescine used herein) the situation is more or less the same as that with H_2_O_2_. Polyamine, with its auto oxidation, may generate a ROS like H_2_O_2_, which might interfere with ripening related activities [17]. Therefore, in comparison, silver would not be a better choice to delay ripening as far as the lycopene and carotenoid contents are concerned. As discussed, ROS is an important moiety in the developmental processes of plants, including fruit ripening [18]. In the present experiment, regardless of the chili variety, a fair accumulation of O_2_^-^ supported the sensitivity in different chemical treatments to the ripening process. The maximum activity of O_2_^-^ was recorded at the ripening period, along with other factors like H_2_O_2_, putrescine, and AgNO_3_. For *cv*. Tejaswani chilies, the role of putrescine in the metabolism of O_2_^-^ may be due to the auto-oxidation of poly amine in the generation of H_2_O_2._ The latter may also be converted into other free radicals like OH^-^. Polyamines, though relevant to stress tolerance, would are ROS producers, on account of their auto-oxidation abilities [19]. The activity of ROS in other gene induction related to cell wall bound peroxidase has been a feature for climacteric fruit ripening [20]. As a consequence of ROS accumulation, oxidized bio-metabolites are hazardous for tissues, as they initiate more free radical generation. In the present experiment, regardless of the duration, the fruit experienced a significant accumulation of lipid peroxides, variably with H_2_O_2_, putrescine, and AgNO_3_ treatments. Upon observation of the nonlinearity between O_2_^-^ and lipid peroxides, OH^-^ assumed the other pathways for free radical generation. ROS generation and its products are common consequences of plant developmental phases, including fruits maturation [21]. In the latter periods of ripening, *cv*. Bullet and *cv*. Tejaswani experienced a significant accumulation of lipid peroxide, even without any chemical treatments. These indicate that the ripening process is ROS dependent, irrespective of any chemical elicitors related to oxidative exposure. Among the few indigenous moieties which inhibit or alter the ripening process, phenolics and their derivatives are the most important. If ROS accelerates or induces processed, then it must be reversed by some other things which quench the intrinsic energy of ROS [22]. Flanonoids, with their various glycosidic assemblages, have anti-oxidation abilities in macromolecules [23]. For total phenolics, putrescine was able to maintain the phenolic content for both cultivars; silver was the next most effective elicitor for the Tejaswani chili variety. This is a genotypic variation which depends upon an elicitor for the same metabolic bio-accumulation. Nevertheless, polyamine is universal for its compatibility with phenolic metabolism through any of its biosynthetic pathways. Therefore, apart from H_2_O_2_ and silver, polyamine could be used for the delayed ripening of fruit. This has been illustrated in fruits where polyamines moderated ripening-specific degenerative processes [24]. One of the most important determinants of fruit ripening is the activity of cell wall hydrolyzing enzymes [25,26]. Thus, we see the expression of cell wall softening enzymes like PME recording the most variability in the early ripening period, but this later becomes inconsistent. Therefore, fruits are expected to receive impulses from H_2_O_2,_ putrescine and AgNO_3_ as elicitors. It is interesting to note that H_2_O_2_ suppressed the activity of PME, and that AgNO_3_ sustained the activity, regardless of the fruit type (*cv*. Bullet and *cv*. Tejaswani). This assumes the ability of H_2_O_2_ as a ROS to down regulate PME expression, whereas AgNO_3_ remains insignificant. Briefly, the pigment content of the fruit skin, secondary metabolites (as phenolics) and cell wall softening-related enzyme activity were significantly affected by chemical elicitation. This statement is most aligned with the findings on day 7 under all the treatments. This is because any fruit which sustains its metabolites after 7 days of storage by suppressing any loss or turn over causing decay is preferred. The behavior of ripening is under genotypic control for any chemical elicitors or abiotic factors. So, the selection of such elicitation may not be precise, but subjected to chili variety, in which flexibility in response to elicitors and maximum utilization in modulation would be the selection criteria.

## 4. Materials and Methods

### 4.1. Plant Materials and Treatments

The present experiment was conducted in the Laboratory of Plant Physiology and Biochemistry, Department of Botany, University of Kalyani. Two varieties of chili, i.e., short-round (*cv*. Bullet) and long-slender (*cv.* Tejaswani), were collected (plant age: 90 days) from horticultural farms at the Agricultural University after maturation into uniform sizes. The collected fruits were checked for any disease or defects, disinfected with a sodium hypochlorite solution, and thoroughly washed with deionized water. The sanitized fruits were divided into four sets according to the chemical treatments used: control (distilled water), H_2_O_2_ (40 mM), putrescine (1 mM), and AgNO_3_ (40µM). Those concentrations caused the minimum decay and loss of freshness of the fruits throughout the maximum duration of the experiment for both the chili varieties. All the treatment sets were transferred to growth chambers which were kept at 35–37 °C, 90–98% relative humidity, and under a light intensity of 5000 Lux. Under these conditions, the fruits were kept for 3, 5, and 7 days; the solutions were changed every other day. Samplings were made after treatment on days 3, 5, and 7 day of incubation. Three replicates (30 fruits each) per treatment were used. All samples were frozen in liquid nitrogen and stored in –80 °C until use in further biochemical assays.

### 4.2. Estimation of Carbohydrate Content

Fruits from different treatments were crushed into fine powders under cold conditions. The reducing sugar was extracted in 70% hot ethanol following repeated boiling, concentrated in a water bath and to reduce the sugar, and 2,4-dinitrosalicylic acid (DNS) reagent was added with crystalline phenol in the ratio of 5:1 dissolved in a 1% NaOH solution. The color was reduced by adding 40% Rochelle salt (Potassium sodium tartrate), and the absorbance was read at 520 nm [27].

For total carbohydrate detection, 500 mg of fruit tissue was taken in different test tubes containing 5.0 mL of 2.5 N HCl, and hydrolyzed for 3 h in a boiling water bath. After cooling, the solution was made neutral by the addition of sodium carbonate powder. The extract was centrifuged at 10,000 ×g for 10 min and the supernatant was collected. The reaction mixture was made by mixing the aliquot with 0.2% anthrone reagent in an acidic state under cold conditions. The reaction mixture was kept in a boiling water bath for 30 min and the color was read at 630 nm [28]. The concentration of sugars was calculated using the standards with dextrose, and expressed on a fresh weight basis.

### 4.3. Estimation of Total Lycopene and Carotenoid Content

First, 0.5 g of material from each sample was crushed into a fine powder and saponified by the addition of 2.5 mL 1.2% alcoholic potassium hydroxide. The saponified extract was mixed with 10.0 mL petroleum ether (AR grade) in a separating funnel and mixed. Upon separation, the aqueous layer was discarded and the upper pigment containing layer was saved. The process continued until the lower layer became transparent, at which time the final volumes for each sample were pulled. The absorbance was read at 450 nm and 503 nm against petroleum ether as the blank. The amount of lycopene and carotenoids were measured using the technique outlined in [29].

Amount of total lyopene = (3.12×A_503_×volume of the samplesolution×100)/weight of the sample (1)

Amount of total carotenoid = (3.12×A_450_×*volume of the samplesolution*×100×4)/*weight of the sample* (2)

### 4.4. Estimation of Total Phenolic and Flavonoid Content

The extraction made in an alcoholic solution and an aliquot of 0.1 mL were mixed thoroughly with Folin-Ciocalteu reagent, followed by the addition of 20% (w/v) Na_2_CO_3_. After incubation of the mixture at 37 °C, the absorbance was recorded at 750 nm. Gallic acid was used and the total content of phenolics was expressed as equivalence of gallic acid. Flavonoid content was estimated; 1.0 g fresh sample was thoroughly crushed and extracted in 50% aqueous methanol. The mixture was concentrated in a water bath, and centrifuged at 8000 ×g at 4 °C for 10 min. To the solution, 10% AlCl_3_ was added. The reaction was checked by adding 1.0 mL 1 M NaOH and further diluted with ethanol. The absorbance was read at 510 nm. The flavonoid content was estimated from the calibration curve with gallic acid.

### 4.5. Detection of H_2_O_2_, O_2_^-^, and Lipid Peroxide

To determine the H_2_O_2_ content,1 g of sample from each treatment was crushed in a 0.01 M phosphate buffer (pH 7.0), and the homogenate was centrifuged at 12,000 ×g for 15 min. Then, the supernatant was mixed with a chromate reagent (5% chromate: glacial acetic acid = 3:1) and kept in a boiling water bath for 10 min. After the development of a green color, the absorbance of the solution was measured at 570 nm. The result was expressed in µM H2O2 g^−1^ fresh weight. For the detection of O_2_^-^, fresh samples were thoroughly crushed in a 65 mM phosphate buffer with a pH of 6.5. The homogenate was centrifuged at 12,000×g for 15 min at 4 °C. The supernatant was rediluted with 65 mM phosphate buffer with a pH of 7.8, to which 10 mM hydroxylamine hydrochloride was added. The mixture was then incubated at 25 °C for 30 min. To the mixture, 10 mM sulfanilamide and 7 Mm alfa-napthylamine were added; this was kept at 37 °C for 20 min. The change of absorbance was recorded taking a reagent blank at 530 nm. The content of O_2_^-^ was estimated from the NaNO_2_ calibration curve, and the extraction coefficient of O_2_^-^as 12.8 mM^−1^ cm^−1^. Then, 1.0 g tissue was thoroughly crushed in 65 mM phosphate buffer (pH 6.5) followed by deprotenization with 0.1% cold TCA. The supernatant was collected by centrifugation at 12,000×g for 10 min at 4 °C. A reaction mixture of 0.5% thiobarbituric acid (TBA) in 20% (w/v) TCA was added. After incubation at 95 °C for 30 min followed by cooling for 1 h, the precipitation was discarded by centrifugation at 10,000×g for 5 min. The supernatant was saved, read at 532 nm and 600 nm, and expressed malondialdehyde (MDA) content/g-fresh weight [30].

### 4.6. Assay of Pectin Methylesterase (PME)

First, 1.0 g of fresh sample was thoroughly crushed into a fine powder, added to liquid N_2_, and homogenized with a buffer of 2.25 gm NaCl-EDTA salt. It was centrifuged 10,000 ×g for 15 min at 4 °C. The supernatant was collected and the residues were re-extracted with the same buffer for 15 min. The supernatant was collected and dissolved in 100 mL cold ethanol with 1.5 mL NaCl. For the reaction, a 1% pectin solution in 5.0 mL 1 M NaCl was used to adjust the pH to 7.5. Then, 0.5 mL enzyme extract was added, followed by the continuous addition of 0.1 N NaOH for 1 h. From a blank of heat-killed enzyme, the alkali consumption was recorded over the time. The activity of the enzymes, expressed as units/mL, was recorded following the method described in [31].

### 4.7. Quantification of Silver Content

One gram of sample was digested in 5 ml triacid mixture (i.e., 98% Sulfuric acid: 69% Nitric acid: 70% Perchloric acid = 3:3:1) at 200 °C for 1 min until the solution becomes clear. Digested samples were diluted 10 times, and the quantification of silver was then done by atomic absorption spectroscopy (PerkinElmer Optima 3300XL, Midland, Canada) [32]. The silver content was expressed as mg/g of plant dry weight.

### 4.8. Statistical Analysis

A student’s t- test was computed using Microsoft Excel to determine significant differences among treatments at *p* ≤ 0.001, *p* ≤ 0.01 and *p* ≤ 0.05 levels of significance. To detect the variations between the two chili varieties under the three treatments (i.e., H_2_O_2_, putrescine, AgNO_3_) as compared to the control, Analysis of Variance (ANOVA), followed by LSD, was calculated using the IBM-SPSS software (Armonk, New York, USA). The Tables for ANOVA may be found in the Supplementary section.

## 5. Conclusions

Fruit ripening is a collective degenerative process. Significant physiological responses in fruit were examined under the application of H_2_O_2_, putrescine, and silver. During postharvest storage, fruits received a significant amount of ROS with a differential bioaccumulation of silver. The variability of silver accumulation would be a selection pressure for cultivar sensitivity to metals. On the other hand, a high accumulation of ROS in pulp tissues might have assisted in the regulation of ripening under the various treatments. For oxidative damages, fruits were more relieved with silver, although this was not the case for all the cultivars. Therefore, the choice of treatment in terms of the antioxidation of metabolites might determine possible storage ability. The retention of metabolites like complex carbohydrates and pigments appeared to be sensitive to all the treatments. The secondary metabolites may be other criteria which could increase the shelf life of fruit when treated with polyamines as well as H_2_O_2_.Interestingly, cell wall loosening activity and the maintenance of redox would meet the regulations of postharvest storage, more so than with silver. Silver apparently showed some advantages over other treatments for its retention of the visual characteristics of chili fruits undergoing delayed ripening. Additionally, putrescine would be next in line in extending shelf life in postharvest storage. Apart from laboratory-based experiments, the use of silver and other chemicals needs to be examined on plants in natural/field conditions.

## Supplementary Materials

The following are available online at https://www.mdpi.com/2223-7747/9/2/238/s1, **Table S1.** Lycopene content. **Table S2.** Total Carotenoid content. **Table S3.** Total Sugar Content. **Table S4:** Total Reducing Sugar content. **Table** **S****5.** Changes of total O^2-^ content. **Table S6.** Changes of total MDA content. **Table S7.** Changes of total phenolic content. **Table S8.** Changes of total Flavonoid Content. Table **S9.** Assay of Pectin Methylesterase. **Table S10.** Silver Content.
