# Supplementary material for: Silver Can Induce Oxidative Stress in Parallel to Other Chemical Elicitors to Modulate the Ripening of Chili Cultivars
Source: Plants (Basel). 2020 Feb 12;9(2):238. doi: 10.3390/plants9020238 (PMC7076538; doi:10.3390/plants9020238)
Supplement: Supplementary file 1 [file plants-09-00238-s001.pdf]

Supplementary Materials

Table S1. Lycopene content.

3 Days

ANOVA

Source of Variation	*SS*	*df*	*MS*	*F*	*P-value*	*F crit*
Variety	59.66107	1	59.66107	9.815999	0.006419	4.493998
Treatments	12485.54	3	4161.848	684.7463	4.23×10^{-17}	3.238872
Interaction	9155.776	3	3051.925	502.1314	4.95×10^{-16}	3.238872
Within	97.24707	16	6.077942			
Total	21798.23	23				

Multiple Comparisons for Tejaswani Chilli Variety
Dependent Variable:
LSD

(I) VAR00001		Mean Difference (I-J)	Std. Error	Sig.	95% Confidence Interval Lower Bound	95% Confidence Interval Upper Bound
$AgNO_3$	Control	-36.9433*	1.86070	0.000	-41.2341	-32.6525
	H_2O_2	5.9000*	1.86070	0.013	1.6092	10.1908
	Putrescine	-100.1000*	1.86070	0.000	-104.3908	-95.8092
Control	$AgNO_3$	36.9433*	1.86070	0.000	32.6525	41.2341
	H_2O_2	42.8433*	1.86070	0.000	38.5525	47.1341

5 Days

ANOVA

Source of Variation	*SS*	*df*	*MS*	*F*	*P-value*	*F crit*
Variety	9881.636	1	9881.636	982.6004	8.59E-16	4.493998
Treatments	4778.191	3	1592.73	158.3764	4.17E-12	3.238872
Interaction	21168.05	3	7056.017	701.6293	3.49E-17	3.238872
Within	160.9059	16	10.05662			
Total	35988.78	23				

Multiple Comparisons for Tejaswani Chilli Variety
Dependent Variable:
LSD

(I) VAR00001		Mean Difference (I-J)	Std. Error	Sig.	95% Confidence Interval Lower Bound	95% Confidence Interval Upper Bound
$AgNO_3$	Control	-73.8267*	2.93455	0.000	-80.5937	-67.0596
	H_2O_2	13.6000*	2.93455	0.002	6.8329	20.3671
	Putrescine	-80.0467*	2.93455	0.000	-86.8137	-73.2796
Control	$AgNO_3$	73.8267*	2.93455	0.000	67.0596	80.5937
	H_2O_2	87.4267*	2.93455	0.000	80.6596	94.1937

7 Days

ANOVA

Source of Variation	*SS*	*df*	*MS*	*F*	*P-value*	*F crit*
Variety	149.0515	1	149.0515	13.6486	0.001965	4.493998
Treatments	80649.37	3	26883.12	2461.679	1.59×10^{-21}	3.238872
Interaction	13241.4	3	4413.8	404.1702	2.75×10^{-15}	3.238872
Within	174.7303	16	10.92065			
Total	94214.55	23				

Multiple Comparisons for Tejaswani Chilli Variety
Dependent Variable:
LSD

(I) VAR00001		Mean Difference (I-J)	Std. Error	Sig.	95% Confidence Interval Lower Bound	95% Confidence Interval Upper Bound
$AgNO_3$	Control	-118.2167*	2.70941	0.000	-124.4646	-111.9688
	H_2O_2	-125.7433*	2.70941	0.000	-131.9912	-119.4954
	Putrescine	-136.3867*	2.70941	0.000	-142.6346	-130.1388
Control	$AgNO_3$	118.2167*	2.70941	0.000	111.9688	124.4646
	H_2O_2	-7.5267*	2.70941	0.024	-13.7746	-1.2788

	Putrescine	-63.1567*	1.86070	0.000	-67.4475	-58.8659
H2O2	AgNO3	-5.9000*	1.86070	0.013	-10.1908	-1.6092
	Control	-42.8433*	1.86070	0.000	-47.1341	-38.5525
	Putresc	-106.0000*	1.86070	0.000	-110.2908	-101.7092
Putrescine	AgNO3	100.1000*	1.86070	0.000	95.8092	104.3908
	Control	63.1567*	1.86070	0.000	58.8659	67.4475
	H2O2	106.0000*	1.86070	0.000	101.7092	110.2908

Based on observed means.
The error term is Mean Square(Error) = 5.193.
*. The mean difference is significant at the 0.05 level.

Multiple Comparisons for Bullet Chilli Variety

Dependent Variable:
LSD

(I) VAR00001		Mean Difference (I-J)	Std. Error	Sig.	95% Confidence Interval Lower Bound	95% Confidence Interval Upper Bound
AgNO3	Control	-0.3067	2.15446	0.890	-5.2749	4.6615
	H2O2	-6.1967*	2.15446	0.021	-11.1649	-1.2285
	Putrescine	-11.7867*	2.15446	0.001	-16.7549	-6.8185
Control	AgNO3	0.3067	2.15446	0.890	-4.6615	5.2749
	H2O2	-5.8900*	2.15446	0.026	-10.8582	-0.9218
	Putrescine	-11.4800*	2.15446	0.001	-16.4482	-6.5118
H2O2	AgN	6.1967	2.154	0.021	1.228	11.16

	Putrescine	-6.2200	2.93455	0.067	-12.9871	0.5471
H2O2	AgNO3	-13.6000*	2.93455	0.002	-20.3671	-6.8329
	Control	-87.4267*	2.93455	0.000	-94.1937	-80.6596
	Putrescine	-93.6467*	2.93455	0.000	-100.4137	-86.8796
Putrescine	AgNO3	80.0467*	2.93455	0.000	73.2796	86.8137
	Control	6.2200	2.93455	0.067	-0.5471	12.9871
	H2O2	93.6467*	2.93455	0.000	86.8796	100.4137

Based on observed means.
The error term is Mean Square(Error) = 12.917.
*. The mean difference is significant at the 0.05 level.

Multiple Comparisons for Bullet Chilli Variety

Dependent Variable:
LSD

(I) VAR00001		Mean Difference (I-J)	Std. Error	Sig.	95% Confidence Interval Lower Bound	95% Confidence Interval Upper Bound
AgNO3	Control	43.8633*	2.19026	0.000	38.8126	48.9141
	H2O2	25.2533*	2.19026	0.000	20.2026	30.3041
	Putrescine	50.2600*	2.19026	0.000	45.2092	55.3108
Control	AgNO3	-43.8633*	2.19026	0.000	-48.9141	-38.8126
	H2O2	-18.6100*	2.19026	0.000	-23.6608	-13.5592
	Putrescine	6.3967*	2.19026	0.019	1.3459	11.4474
H2O2	AgN	-25.25	2.190	0.000	-30.3	-20.2

	Putrescine	-18.1700*	2.70941	0.000	-24.4179	-11.9221
H2O2	AgNO3	125.7433*	2.70941	0.000	119.4954	131.9912
	Control	7.5267*	2.70941	0.024	1.2788	13.7746
	Putrescine	-10.6433*	2.70941	0.004	-16.8912	-4.3954
Putrescine	AgNO3	136.3867*	2.70941	0.000	130.1388	142.6346
	Control	18.1700*	2.70941	0.000	11.9221	24.4179
	H2O2	10.6433*	2.70941	0.004	4.3954	16.8912

Based on observed means.
The error term is Mean Square(Error) = 11.011.
*. The mean difference is significant at the 0.05 level.

Multiple Comparisons for Bullet Chilli Variety

Dependent Variable:
LSD

(I) VAR00001		Mean Difference (I-J)	Std. Error	Sig.	95% Confidence Interval Lower Bound	95% Confidence Interval Upper Bound
AgNO3	Control	-168.2700*	2.68700	0.000	-174.4662	-162.0738
	H2O2	-155.2933*	2.68700	0.000	-161.4896	-149.0971
	Putrescine	-62.5133*	2.68700	0.000	-68.7096	-56.3171
Control	AgNO3	168.2700*	2.68700	0.000	162.0738	174.4662
	H2O2	12.9767*	2.68700	0.001	6.7804	19.1729
	Putrescine	105.7567*	2.68700	0.000	99.5604	111.9529
H2O2	AgN	155.29	2.687	0.000	149.0	161.4

	O3	*	46		5	49
	Control	5.8900*	2.15446	0.026	0.9218	10.8582
	Putresc	-5.5900*	2.15446	0.032	-10.5582	-0.6218
Putrescine	AgNO3	11.7867*	2.15446	0.001	6.8185	16.7549
	Control	11.4800*	2.15446	0.001	6.5118	16.4482
	H2O2	5.5900*	2.15446	0.032	0.6218	10.5582

Based on observed means.
The error term is Mean Square(Error) = 6.963.
*. The mean difference is significant at the 0.05 level.

	O3	33*	26		041	026
	Control	18.6100*	2.19026	0.000	13.5592	23.6608
	Putrescine	25.0067*	2.19026	0.000	19.9559	30.0574
Putrescine	AgNO3	-50.2600*	2.19026	0.000	-55.3108	-45.2092
	Control	-6.3967*	2.19026	0.019	-11.4474	-1.3459
	H2O2	-25.0067*	2.19026	0.000	-30.0574	-19.9559

Based on observed means.
The error term is Mean Square(Error) = 7.196.
*. The mean difference is significant at the 0.05 level.

	O3	33*	00		971	896
	Control	-12.9767*	2.68700	0.001	-19.1729	-6.7804
	Putrescine	92.7800*	2.68700	0.000	86.5838	98.9762
Putrescine	AgNO3	62.5133*	2.68700	0.000	56.3171	68.7096
	Control	-105.7567*	2.68700	0.000	-111.9529	-99.5604
	H2O2	-92.7800*	2.68700	0.000	-98.9762	-86.5838

Based on observed means.
The error term is Mean Square(Error) = 10.830.
*. The mean difference is significant at the 0.05 level.

Table S2. Total Carotenoid content.

3 Days

ANOVA

Source of Variation	*SS*	*df*	*MS*	*F*	*P-value*	*F crit*
Variety	247.0417	1	247.0417	2.195926	0.157802	4.493998
Treatments	258212.5	3	86070.82	765.074	1.76E-17	3.238872
Interaction	140779.5	3	46926.49	417.1243	2.14E-15	3.238872
Within	1800	16	112.5			
Total	401039	23				

5 Days

ANOVA

Source of Variation	*SS*	*df*	*MS*	*F*	*P-value*	*F crit*
Variety	222915.4	1	222915.4	1364.092	6.44E-17	4.493998
Treatments	57294.46	3	19098.15	116.8678	4.31E-11	3.238872
Interaction	312786.1	3	104262	638.0135	7.42E-17	3.238872
Within	2614.667	16	163.4167			
Total	595610.6	23				

7 Days

ANOVA

Source of Variation	*SS*	*df*	*MS*	*F*	*P-value*	*F crit*
Variety	415277	1	415277	684.946	1.46E-14	4.493998
Treatments	2113275	3	704424.9	1161.858	6.33E-19	3.238872
Interaction	487855.5	3	162618.5	268.2182	6.91E-14	3.238872
Within	9700.667	16	606.2917			
Total	3026108	23				

Multiple Comparisons for Tejaswani Chilli Variety (3 Days)

Dependent Variable: LSD

(I) VAR00001		Mean Difference (I-J)	Std. Error	Sig.	95% Confidence Interval Lower Bound	95% Confidence Interval Upper Bound
AgNO3	Control	-255.6667*	8.45577	0.000	-275.1657	-236.1676
	H2O2	-79.3333*	8.45577	0.000	-98.8324	-59.8343
	Putrescine	-368.0000*	8.45577	0.000	-387.4990	-348.5010
Control	AgNO3	255.6667*	8.45577	0.000	236.1676	275.1657
	H2O2	176.3333*	8.45577	0.000	156.8343	195.8324
	Putrescine	-112.3333*	8.45577	0.000	-131.8324	-92.8343
H2O2	AgNO3	79.3333*	8.45577	0.000	59.8343	98.8324

Multiple Comparisons for Tejaswani Chilli Variety (5 Days)

Dependent Variable: LSD

(I) VAR00001		Mean Difference (I-J)	Std. Error	Sig.	95% Confidence Interval Lower Bound	95% Confidence Interval Upper Bound
AgNO3	Control	-210.0000*	12.67544	0.000	-239.2296	-180.7704
	H2O2	-66.6667*	12.67544	0.001	-95.8963	-37.4371
	Putrescine	-320.0000*	12.67544	0.000	-349.2296	-290.7704
Control	AgNO3	210.0000*	12.67544	0.000	180.7704	239.2296
	H2O2	143.3333*	12.67544	0.000	114.1037	172.5629
	Putrescine	-110.0000*	12.67544	0.000	-139.2296	-80.7704
H2O2	AgNO3	66.6667*	12.67544	0.001	37.4371	95.8963

Multiple Comparisons for Tejaswani Chilli Variety (7 Days)

Dependent Variable: LSD

(I) VAR00001		Mean Difference (I-J)	Std. Error	Sig.	95% Confidence Interval Lower Bound	95% Confidence Interval Upper Bound
AgNO3	Control	-937.3333*	18.62495	0.000	-980.2826	-894.3841
	H2O2	-331.3333*	18.62495	0.000	-374.2826	-288.3841
	Putrescine	-839.3333*	18.62495	0.000	-882.2826	-796.3841
Control	AgNO3	937.3333*	18.62495	0.000	894.3841	980.2826
	H2O2	606.0000*	18.62495	0.000	563.0508	648.9492
	Putrescine	98.0000*	18.62495	0.001	55.0508	140.9492
H2O2	AgNO3	331.3333*	18.62495	0.000	288.3841	374.2826

	Control	-176.3333*	8.45577	0.000	-195.8324	-156.8343
	Putrescine	-288.6667*	8.45577	0.000	-308.1657	-269.1676
Putrescine	AgNO3	368.0000*	8.45577	0.000	348.5010	387.4990
	Control	112.3333*	8.45577	0.000	92.8343	131.8324
	H2O2	288.6667*	8.45577	0.000	269.1676	308.1657

Based on observed means.
The error term is Mean Square(Error) = 107.250.
*. The mean difference is significant at the 0.05 level.

Multiple Comparisons for Bullet Chilli Variety

Dependent Variable:
LSD

(I) VAR00001		Mean Difference (I-J)	Std. Error	Sig.	95% Confidence Interval	
					Lower Bound	Upper Bound
AgNO3	Control	-31.3333*	8.86002	0.008	-51.7646	-10.9021
	H2O2	-254.3333*	8.86002	0.000	-274.7646	-233.9021
	Putrescine	-216.3333*	8.86002	0.000	-236.7646	-195.9021
Control	AgNO3	31.3333*	8.86002	0.008	10.9021	51.7646
	H2O2	-223.0000*	8.86002	0.000	-243.4312	-202.5688
	Putrescine	-185.0000*	8.86002	0.000	-205.4312	-164.5688
H2O2	AgNO3	254.3333*	8.86002	0.000	233.9021	274.7646
	Control	223.0000*	8.86002	0.000	202.5688	243.4312
	Putres	38.000	8.860	0.003	17.56	58.43

	Control	-143.3333*	12.67544	0.000	-172.5629	-114.1037
	Putrescine	-253.3333*	12.67544	0.000	-282.5629	-224.1037
Putrescine	AgNO3	320.0000*	12.67544	0.000	290.7704	349.2296
	Control	110.0000*	12.67544	0.000	80.7704	139.2296
	H2O2	253.3333*	12.67544	0.000	224.1037	282.5629

Based on observed means.
The error term is Mean Square(Error) = 241.000.
*. The mean difference is significant at the 0.05 level.

Multiple Comparisons for Bullet Chilli Variety

Dependent Variable:
LSD

(I) VAR00001		Mean Difference (I-J)	Std. Error	Sig.	95% Confidence Interval	
					Lower Bound	Upper Bound
AgNO3	Control	-49.3333*	7.56454	0.000	-66.7772	-31.8895
	H2O2	-137.0000*	7.56454	0.000	-154.4439	-119.5561
	Putrescine	200.6667*	7.56454	0.000	183.2228	218.1105
Control	AgNO3	49.3333*	7.56454	0.000	31.8895	66.7772
	H2O2	-87.6667*	7.56454	0.000	-105.1105	-70.2228
	Putrescine	250.0000*	7.56454	0.000	232.5561	267.4439
H2O2	AgNO3	137.0000*	7.56454	0.000	119.5561	154.4439
	Control	87.6667*	7.56454	0.000	70.2228	105.1105
	Putres	337.66	7.564	0.000	320.2	355.1

	Control	-606.0000*	18.62495	0.000	-648.9492	-563.0508
	Putrescine	-508.0000*	18.62495	0.000	-550.9492	-465.0508
Putrescine	AgNO3	839.3333*	18.62495	0.000	796.3841	882.2826
	Control	-98.0000*	18.62495	0.001	-140.9492	-55.0508
	H2O2	508.0000*	18.62495	0.000	465.0508	550.9492

Based on observed means.
The error term is Mean Square(Error) = 520.333.
*. The mean difference is significant at the 0.05 level.

Multiple Comparisons for Bullet Chilli Variety

Dependent Variable:
LSD

(I) VAR00001		Mean Difference (I-J)	Std. Error	Sig.	95% Confidence Interval	
					Lower Bound	Upper Bound
AgNO3	Control	-715.0000*	21.48255	0.000	-764.5389	-665.4611
	H2O2	-448.0000*	21.48255	0.000	-497.5389	-398.4611
	Putrescine	-208.6667*	21.48255	0.000	-258.2055	-159.1278
Control	AgNO3	715.0000*	21.48255	0.000	665.4611	764.5389
	H2O2	267.0000*	21.48255	0.000	217.4611	316.5389
	Putrescine	506.3333*	21.48255	0.000	456.7945	555.8722
H2O2	AgNO3	448.0000*	21.48255	0.000	398.4611	497.5389
	Control	-267.0000*	21.48255	0.000	-316.5389	-217.4611
	Putres	239.33	21.48	0.000	189.7	288.8

	cine	0*	02		88	12		cine	67*	54		228	105		cine	33*	255		945	722
Putrescine	AgNO3	216.3333*	8.86002	0.000	195.9021	236.7646	Putrescine	AgNO3	-200.6667*	7.56454	0.000	-218.1105	-183.2228	Putrescine	AgNO3	208.6667*	21.48255	0.000	159.1278	258.2055
	Control	185.0000*	8.86002	0.000	164.5688	205.4312		Control	-250.0000*	7.56454	0.000	-267.4439	-232.5561		Control	-506.3333*	21.48255	0.000	-555.8722	-456.7945
	H2O2	-38.0000*	8.86002	0.003	-58.4312	-17.5688		H2O2	-337.6667*	7.56454	0.000	-355.1105	-320.2228		H2O2	-239.3333*	21.48255	0.000	-288.8722	-189.7945

Based on observed means.
The error term is Mean Square(Error) = 117.750.
*. The mean difference is significant at the 0.05 level.

Based on observed means.
The error term is Mean Square(Error) = 85.833.
*. The mean difference is significant at the 0.05 level.

Based on observed means.
The error term is Mean Square(Error) = 692.250.
*. The mean difference is significant at the 0.05 level.

Table S3. Total Sugar Content.

3 Days

ANOVA

Source of Variation	*SS*	*df*	*MS*	*F*	*P-value*	*F crit*
Variety	116343.4	1	116343.4	4211.525	8.28E-21	4.493998
Treatments	45415.13	3	15138.38	547.9955	2.48E-16	3.238872
Interaction	125269.1	3	41756.38	1511.543	7.78E-20	3.238872
Within	442	16	27.625			
Total	287469.6	23				

5 Days

ANOVA

Source of Variation	*SS*	*df*	*MS*	*F*	*P-value*	*F crit*
Variety	110976	1	110976	2706.732	2.8E-19	4.493998
Treatments	109803	3	36601	892.7073	5.15E-18	3.238872
Interaction	74259	3	24753	603.7317	1.15E-16	3.238872
Within	656	16	41			
Total	295694	23				

7 Days

ANOVA

Source of Variation	*SS*	*df*	*MS*	*F*	*P-value*	*F crit*
Variety	337.5	1	337.5	17.08861	0.000779	4.493998
Treatments	15972	3	5324	269.5696	6.64E-14	3.238872
Interaction	43264.5	3	14421.5	730.2025	2.54E-17	3.238872
Within	316	16	19.75			
Total	59890	23				

Multiple Comparisons for Tejaswani Chilli Variety (3 Days)
Dependent Variable:
LSD

(I) VAR00001		Mean Difference (I-J)	Std. Error	Sig.	95% Confidence Interval Lower Bound	95% Confidence Interval Upper Bound
AgNO3	Control	-17.0000*	3.24037	0.001	-24.4723	-9.5277
	H2O2	106.0000*	3.24037	0.000	98.5277	113.4723
	Putresc	83.0000*	3.24037	0.000	75.5277	90.4723
Control	AgNO3	17.0000*	3.24037	0.001	9.5277	24.4723
	H2O2	123.0000*	3.24037	0.000	115.5277	130.4723
	Putresc	100.0000*	3.24037	0.000	92.5277	107.4723
H2O2	AgN	-106.0	3.240	0.000	-113.	-98.5

Multiple Comparisons for Tejaswani Chilli Variety (5 Days)
Dependent Variable:
LSD

(I) VAR00001		Mean Difference (I-J)	Std. Error	Sig.	95% Confidence Interval Lower Bound	95% Confidence Interval Upper Bound
AgNO3	Control	35.0000*	4.77842	0.000	23.9809	46.0191
	H2O2	0.0000	4.77842	1.000	-11.0191	11.0191
	Putresc	193.0000*	4.77842	0.000	181.9809	204.0191
Control	AgNO3	-35.0000*	4.77842	0.000	-46.0191	-23.9809
	H2O2	-35.0000*	4.77842	0.000	-46.0191	-23.9809
	Putresc	158.0000*	4.77842	0.000	146.9809	169.0191
H2O2	AgN	0.0000	4.77	1.000	-11.0	11.01

Multiple Comparisons for Tejaswani Chilli Variety (7 Days)
Dependent Variable:
LSD

(I) VAR00001		Mean Difference (I-J)	Std. Error	Sig.	95% Confidence Interval Lower Bound	95% Confidence Interval Upper Bound
AgNO3	Control	-105.0000*	3.74166	0.000	-113.6283	-96.3717
	H2O2	-135.0000*	3.74166	0.000	-143.6283	-126.3717
	Putresc	-45.0000*	3.74166	0.000	-53.6283	-36.3717
Control	AgNO3	105.0000*	3.74166	0.000	96.3717	113.6283
	H2O2	-30.0000*	3.74166	0.000	-38.6283	-21.3717
	Putresc	60.0000*	3.74166	0.000	51.3717	68.6283
H2O2	AgN	135.00	3.74	0.000	126.3	143.6

	O3	000*	37		4723	277
	Control	-123.0000*	3.24037	0.000	-130.4723	-115.5277
	Putresc	-23.0000*	3.24037	0.000	-30.4723	-15.5277
Putrescine	AgNO3	-83.0000*	3.24037	0.000	-90.4723	-75.5277
	Control	-100.0000*	3.24037	0.000	-107.4723	-92.5277
	H2O2	23.0000*	3.24037	0.000	15.5277	30.4723

Based on observed means.
The error term is Mean Square(Error) = 15.750.
*. The mean difference is significant at the 0.05 level.

	O3		842		191	91
	Control	35.0000*	4.77842	0.000	23.9809	46.0191
	Putresc	193.0000*	4.77842	0.000	181.9809	204.0191
Putrescine	AgNO3	-193.0000*	4.77842	0.000	-204.0191	-181.9809
	Control	-158.0000*	4.77842	0.000	-169.0191	-146.9809
	H2O2	-193.0000*	4.77842	0.000	-204.0191	-181.9809

Based on observed means.
The error term is Mean Square(Error) = 34.250.
*. The mean difference is significant at the 0.05 level.

	O3	00*	166		717	283
	Control	30.0000*	3.74166	0.000	21.3717	38.6283
	Putresc	90.0000*	3.74166	0.000	81.3717	98.6283
Putrescine	AgNO3	45.0000*	3.74166	0.000	36.3717	53.6283
	Control	-60.0000*	3.74166	0.000	-68.6283	-51.3717
	H2O2	-90.0000*	3.74166	0.000	-98.6283	-81.3717

Based on observed means.
The error term is Mean Square(Error) = 21.000.
*. The mean difference is significant at the 0.05 level.

Multiple Comparisons for Bullet Chilli Variety
Dependent Variable:
LSD

(I) VAR00001		Mean Difference (I-J)	Std. Error	Sig.	95% Confidence Interval Lower Bound	Upper Bound
AgNO3	Control	263.0000*	5.13160	0.000	251.1665	274.8335
	H2O2	17.0000*	5.13160	0.011	5.1665	28.8335
	Putrescine	35.0000*	5.13160	0.000	23.1665	46.8335
Control	AgNO3	-263.0000*	5.13160	0.000	-274.8335	-251.1665
	H2O2	-246.0000*	5.13160	0.000	-257.8335	-234.1665
	Putrescine	-228.0000*	5.13160	0.000	-239.8335	-216.1665
H2O2	AgNO3	-17.0000*	5.13160	0.011	-28.8335	-5.1665
	Control	246.0000*	5.13160	0.000	234.1665	257.8335

Multiple Comparisons for Bullet Chilli Variety
Dependent Variable:
LSD

(I) VAR00001		Mean Difference (I-J)	Std. Error	Sig.	95% Confidence Interval Lower Bound	Upper Bound
AgNO3	Control	-246.0000*	5.64210	0.000	-259.0107	-232.9893
	H2O2	-176.0000*	5.64210	0.000	-189.0107	-162.9893
	Putrescine	-70.0000*	5.64210	0.000	-83.0107	-56.9893
Control	AgNO3	246.0000*	5.64210	0.000	232.9893	259.0107
	H2O2	70.0000*	5.64210	0.000	56.9893	83.0107
	Putrescine	176.0000*	5.64210	0.000	162.9893	189.0107
H2O2	AgNO3	176.0000*	5.64210	0.000	162.9893	189.0107
	Control	-70.0000*	5.64210	0.000	-83.0107	-56.9893

Multiple Comparisons for Bullet Chilli Variety
Dependent Variable:
LSD

(I) VAR00001		Mean Difference (I-J)	Std. Error	Sig.	95% Confidence Interval Lower Bound	Upper Bound
AgNO3	Control	122.0000*	3.76386	0.000	113.3205	130.6795
	H2O2	104.0000*	3.76386	0.000	95.3205	112.6795
	Putrescine	0.0000	3.76386	1.000	-8.6795	8.6795
Control	AgNO3	-122.0000*	3.76386	0.000	-130.6795	-113.3205
	H2O2	-18.0000*	3.76386	0.001	-26.6795	-9.3205
	Putrescine	-122.0000*	3.76386	0.000	-130.6795	-113.3205
H2O2	AgNO3	-104.0000*	3.76386	0.000	-112.6795	-95.3205
	Control	18.0000*	3.76386	0.001	9.3205	26.6795

	Putrescine	18.0000*	5.13160	0.008	6.1665	29.8335
Putrescine	AgNO3	-35.0000*	5.13160	0.000	-46.8335	-23.1665
	Control	228.0000*	5.13160	0.000	216.1665	239.8335
	H2O2	-18.0000*	5.13160	0.008	-29.8335	-6.1665

Based on observed means.
The error term is Mean Square(Error) = 39.500.
*. The mean difference is significant at the 0.05 level.

	Putrescine	106.0000*	5.64210	0.000	92.9893	119.0107
Putrescine	AgNO3	70.0000*	5.64210	0.000	56.9893	83.0107
	Control	-176.0000*	5.64210	0.000	-189.0107	-162.9893
	H2O2	-106.0000*	5.64210	0.000	-119.0107	-92.9893

Based on observed means.
The error term is Mean Square(Error) = 47.750.
*. The mean difference is significant at the 0.05 level.

	Putrescine	-104.0000*	3.76386	0.000	-112.6795	-95.3205
Putrescine	AgNO3	0.0000	3.76386	1.000	-8.6795	8.6795
	Control	122.0000*	3.76386	0.000	113.3205	130.6795
	H2O2	104.0000*	3.76386	0.000	95.3205	112.6795

Based on observed means.
The error term is Mean Square(Error) = 21.250.
*. The mean difference is significant at the 0.05 level.

Table 4. Total Reducing Sugar content.

3 Days

ANOVA

Source of Variation	*SS*	*df*	*MS*	*F*	*P-value*	*F crit*
Variety	0.429338	1	0.429338	196.2686	2.12E-10	4.493998
Treatments	0.613913	3	0.204638	93.54857	2.33E-10	3.238872
Interaction	0.708413	3	0.236138	107.9486	7.89E-11	3.238872
Within	0.035	16	0.002188			
Total	1.786663	23				

5 Days

ANOVA

Source of Variation	*SS*	*df*	*MS*	*F*	*P-value*	*F crit*
Variety	0.132017	1	0.132017	50.37202	2.53E-06	4.493998
Treatments	0.512017	3	0.170672	65.12136	3.48E-09	3.238872
Interaction	0.720017	3	0.240006	91.57605	2.74E-10	3.238872
Within	0.041933	16	0.002621			
Total	1.405983	23				

7 Days

ANOVA

Source of Variation	*SS*	*df*	*MS*	*F*	*P-value*	*F crit*
Variety	0.113438	1	0.113438	40.51339	9.38E-06	4.493998
Treatments	0.157913	3	0.052638	18.79911	1.7E-05	3.238872
Interaction	1.336913	3	0.445638	159.1563	4.02E-12	3.238872
Within	0.0448	16	0.0028			
Total	1.653063	23				

Multiple Comparisons for Tejaswani Chilli Variety (3 Days)
Dependent Variable: LSD

(I) VAR00001		Mean Difference (I-J)	Std. Error	Sig.	95% Confidence Interval Lower Bound	95% Confidence Interval Upper Bound
AgNO3	Control	.5000*	0.03937	0.000	0.4092	0.5908
	H2O2	.2800*	0.03937	0.000	0.1892	0.3708
	Putrescine	.8800*	0.03937	0.000	0.7892	0.9708
Control	AgNO3	-.5000*	0.03937	0.000	-0.5908	-0.4092
	H2O2	-.2200*	0.03937	0.001	-0.3108	-0.1292
	Putrescine	.3800*	0.03937	0.000	0.2892	0.4708
H2O2	AgN	-.2800*	0.039	0.000	-0.37	-0.18

Multiple Comparisons for Tejaswani Chilli Variety (5 Days)
Dependent Variable: LSD

(I) VAR00001		Mean Difference (I-J)	Std. Error	Sig.	95% Confidence Interval Lower Bound	95% Confidence Interval Upper Bound
AgNO3	Control	0.0533	0.04116	0.231	-0.0416	0.1483
	H2O2	-0.0600	0.04116	0.183	-0.1549	0.0349
	Putrescine	.2733*	0.04116	0.000	0.1784	0.3683
Control	AgNO3	-0.0533	0.04116	0.231	-0.1483	0.0416
	H2O2	-.1133*	0.04116	0.025	-0.2083	-0.0184
	Putrescine	.2200*	0.04116	0.001	0.1251	0.3149
H2O2	AgN	0.0600	0.041	0.183	-0.03	0.154

Multiple Comparisons for Tejaswani Chilli Variety (7 Days)
Dependent Variable: LSD

(I) VAR00001		Mean Difference (I-J)	Std. Error	Sig.	95% Confidence Interval Lower Bound	95% Confidence Interval Upper Bound
AgNO3	Control	.1300*	0.04082	0.013	0.0359	0.2241
	H2O2	-.3600*	0.04082	0.000	-0.4541	-0.2659
	Putrescine	.4300*	0.04082	0.000	0.3359	0.5241
Control	AgNO3	-.1300*	0.04082	0.013	-0.2241	-0.0359
	H2O2	-.4900*	0.04082	0.000	-0.5841	-0.3959
	Putrescine	.3000*	0.04082	0.000	0.2059	0.3941
H2O2	AgN	.3600*	0.040	0.000	0.265	0.454

	O3		37		08	92
	Control	.2200*	0.03937	0.001	0.1292	0.3108
	Putrescine	.6000*	0.03937	0.000	0.5092	0.6908
Putrescine	AgNO3	-.8800*	0.03937	0.000	-0.9708	-0.7892
	Control	-.3800*	0.03937	0.000	-0.4708	-0.2892
	H2O2	-.6000*	0.03937	0.000	-0.6908	-0.5092

Based on observed means.
The error term is Mean Square(Error) =0 .002.
*. The mean difference is significant at the 0.05 level.

Multiple Comparisons for Bullet Chilli Variety

Dependent Variable:
LSD

(I) VAR00001		Mean Difference (I-J)	Std. Error	Sig.	95% Confidence Interval Lower Bound	Upper Bound
AgNO3	Control	-.1200*	0.03697	0.012	-0.2052	-0.0348
	H2O2	.1100*	0.03697	0.018	0.0248	0.1952
	Putrescine	0.0200	0.03697	0.603	-0.0652	0.1052
Control	AgNO3	.1200*	0.03697	0.012	0.0348	0.2052
	H2O2	.2300*	0.03697	0.000	0.1448	0.3152
	Putrescine	.1400*	0.03697	0.005	0.0548	0.2252
H2O2	AgNO3	-.1100*	0.03697	0.018	-0.1952	-0.0248
	Control	-.2300*	0.03697	0.000	-0.3152	-0.1448

	O3		16		49	9
	Control	.1133*	0.04116	0.025	0.0184	0.2083
	Putrescine	.3333*	0.04116	0.000	0.2384	0.4283
Putrescine	AgNO3	-.2733*	0.04116	0.000	-0.3683	-0.1784
	Control	-.2200*	0.04116	0.001	-0.3149	-0.1251
	H2O2	-.3333*	0.04116	0.000	-0.4283	-0.2384

Based on observed means.
The error term is Mean Square(Error) =0 .003.
*. The mean difference is significant at the 0.05 level.

Multiple Comparisons for Bullet Chilli Variety

Dependent Variable:
LSD

(I) VAR00001		Mean Difference (I-J)	Std. Error	Sig.	95% Confidence Interval Lower Bound	Upper Bound
AgNO3	Control	-.4000*	0.04243	0.000	-0.4978	-0.3022
	H2O2	.4300*	0.04243	0.000	0.3322	0.5278
	Putrescine	0.0700	0.04243	0.138	-0.0278	0.1678
Control	AgNO3	.4000*	0.04243	0.000	0.3022	0.4978
	H2O2	.8300*	0.04243	0.000	0.7322	0.9278
	Putrescine	.4700*	0.04243	0.000	0.3722	0.5678
H2O2	AgNO3	-.4300*	0.04243	0.000	-0.5278	-0.3322
	Control	-.8300*	0.04243	0.000	-0.9278	-0.7322

	O3		82		9	1
	Control	.4900*	0.04082	0.000	0.3959	0.5841
	Putrescine	.7900*	0.04082	0.000	0.6959	0.8841
Putrescine	AgNO3	-.4300*	0.04082	0.000	-0.5241	-0.3359
	Control	-.3000*	0.04082	0.000	-0.3941	-0.2059
	H2O2	-.7900*	0.04082	0.000	-0.8841	-0.6959

Based on observed means.
The error term is Mean Square(Error) =0 .002.
*. The mean difference is significant at the 0.05 level.

Multiple Comparisons for Bullet Chilli Variety

Dependent Variable:
LSD

(I) VAR00001		Mean Difference (I-J)	Std. Error	Sig.	95% Confidence Interval Lower Bound	Upper Bound
AgNO3	Control	-.1100*	0.04546	0.042	-0.2148	-0.0052
	H2O2	.4300*	0.04546	0.000	0.3252	0.5348
	Putrescine	-0.0300	0.04546	0.528	-0.1348	0.0748
Control	AgNO3	.1100*	0.04546	0.042	0.0052	0.2148
	H2O2	.5400*	0.04546	0.000	0.4352	0.6448
	Putrescine	0.0800	0.04546	0.116	-0.0248	0.1848
H2O2	AgNO3	-.4300*	0.04546	0.000	-0.5348	-0.3252
	Control	-.5400*	0.04546	0.000	-0.6448	-0.4352

Putrescine	Putrescine	-.0900*	0.03697	0.041	-0.1752	-0.0048	Putrescine	Putrescine	-.3600*	0.04243	0.000	-0.4578	-0.2622	Putrescine	Putrescine	-.4600*	0.04546	0.000	-0.5648	-0.3552
	AgNO3	-0.0200	0.03697	0.603	-0.1052	0.0652		AgNO3	-0.0700	0.04243	0.138	-0.1678	0.0278		AgNO3	0.0300	0.04546	0.528	-0.0748	0.1348
	Control	-.1400*	0.03697	0.005	-0.2252	-0.0548		Control	-.4700*	0.04243	0.000	-0.5678	-0.3722		Control	-0.0800	0.04546	0.116	-0.1848	0.0248
	H2O2	.0900*	0.03697	0.041	0.0048	0.1752		H2O2	.3600*	0.04243	0.000	0.2622	0.4578		H2O2	.4600*	0.04546	0.000	0.3552	0.5648

Based on observed means.
The error term is Mean Square(Error) = 0.002.
*. The mean difference is significant at the 0.05 level.

Based on observed means.
The error term is Mean Square(Error) =0 .003.
*. The mean difference is significant at the 0.05 level.

Based on observed means.
The error term is Mean Square(Error) =0 .003.
*. The mean difference is significant at the 0.05 level.

Table S5. Changes of total O^{2-} conten.

3 Days

ANOVA

Source of Variation	*SS*	*df*	*MS*	*F*	*P-value*	*F crit*
Variety	0.005582	1	0.005582	10.71648	0.004778	4.493998
Treatments	0.689672	3	0.229891	441.3898	1.37E-15	3.238872
Interaction	0.101786	3	0.033929	65.14272	3.47E-09	3.238872
Within	0.008333	16	0.000521			
Total	0.805372	23				

5 Days

ANOVA

Source of Variation	*SS*	*df*	*MS*	*F*	*P-value*	*F crit*
Variety	0.019494	1	0.019494	22.85011	0.000204	4.493998
Treatments	0.120713	3	0.040238	47.16484	3.61E-08	3.238872
Interaction	1.189956	3	0.396652	464.9401	9.09E-16	3.238872
Within	0.01365	16	0.000853			
Total	1.343813	23				

7 Days

ANOVA

Source of Variation	*SS*	*df*	*MS*	*F*	*P-value*	*F crit*
Variety	0.198198	1	0.198198	3103.411	9.43E-20	4.493998
Treatments	0.040963	3	0.013654	213.8016	4.07E-13	3.238872
Interaction	0.029059	3	0.009686	151.6703	5.83E-12	3.238872
Within	0.001022	16	6.39E-05			
Total	0.269242	23				

Multiple Comparisons for Tejaswani Chilli Variety
Dependent Variable: LSD

(I) VAR00001		Mean Difference (I-J)	Std. Error	Sig.	95% Confidence Interval Lower Bound	Upper Bound
AgNO3	Control	-.1060*	0.01760	0.000	-0.1466	-0.0654
	H2O2	-.2370*	0.01760	0.000	-0.2776	-0.1964
	Putrescine	-.4943*	0.01760	0.000	-0.5349	-0.4538
Control	AgNO3	.1060*	0.01760	0.000	0.0654	0.1466
	H2O2	-.1310*	0.01760	0.000	-0.1716	-0.0904
	Putrescine	-.3883*	0.01760	0.000	-0.4289	-0.3478
H2O2	AgN	.2370*	0.017	0.000	0.196	0.277

Multiple Comparisons for Tejaswani Chilli Variety
Dependent Variable: LSD

(I) VAR00001		Mean Difference (I-J)	Std. Error	Sig.	95% Confidence Interval Lower Bound	Upper Bound
AgNO3	Control	-.1630*	0.02291	0.000	-0.2158	-0.1102
	H2O2	-.5450*	0.02291	0.000	-0.5978	-0.4922
	Putrescine	-.5910*	0.02291	0.000	-0.6438	-0.5382
Control	AgNO3	.1630*	0.02291	0.000	0.1102	0.2158
	H2O2	-.3820*	0.02291	0.000	-0.4348	-0.3292
	Putrescine	-.4280*	0.02291	0.000	-0.4808	-0.3752
H2O2	AgN	.5450*	0.022	0.000	0.492	0.597

Multiple Comparisons for Tejaswani Chilli Variety
Dependent Variable: LSD

(I) Treatment		Mean Difference (I-J)	Std. Error	Sig.	95% Confidence Interval Lower Bound	Upper Bound
AgNO3	Control	-.1640*	0.00907	0.000	-0.1849	-0.1431
	H2O2	-.1460*	0.00907	0.000	-0.1669	-0.1251
	Putrescine	-.1730*	0.00907	0.000	-0.1939	-0.1521
Control	AgNO3	.1640*	0.00907	0.000	0.1431	0.1849
	H2O2	0.0180	0.00907	0.082	-0.0029	0.0389
	Putrescine	-0.0090	0.00907	0.350	-0.0299	0.0119
H2O2	AgN	.1460*	0.009	0.000	0.125	0.166

	O3		60		4	6
	Control	.1310*	0.01760	0.000	0.0904	0.1716
	Putrescine	-.2573*	0.01760	0.000	-0.2979	-0.2168
Putrescine	AgNO3	.4943*	0.01760	0.000	0.4538	0.5349
	Control	.3883*	0.01760	0.000	0.3478	0.4289
	H2O2	.2573*	0.01760	0.000	0.2168	0.2979

Based on observed means.
The error term is Mean Square(Error) = 0.000.
*. The mean difference is significant at the 0.05 level.

Multiple Comparisons for Bullet Chilli Variety
Dependent Variable:
LSD

(I) VAR00001		Mean Difference (I-J)	Std. Error	Sig.	95% Confidence Interval Lower Bound	95% Confidence Interval Upper Bound
AgNO3	Control	-.3550*	0.01961	0.000	-0.4002	-0.3098
	H2O2	-.1460*	0.01961	0.000	-0.1912	-0.1008
	Putrescine	-.4583*	0.01961	0.000	-0.5036	-0.4131
Control	AgNO3	.3550*	0.01961	0.000	0.3098	0.4002
	H2O2	.2090*	0.01961	0.000	0.1638	0.2542
	Putrescine	-.1033*	0.01961	0.001	-0.1486	-0.0581
H2O2	AgNO3	.1460*	0.01961	0.000	0.1008	0.1912
	Control	-.2090*	0.01961	0.000	-0.2542	-0.1638

	O3		91		2	8
	Control	.3820*	0.02291	0.000	0.3292	0.4348
	Putrescine	-0.0460	0.02291	0.080	-0.0988	0.0068
Putrescine	AgNO3	.5910*	0.02291	0.000	0.5382	0.6438
	Control	.4280*	0.02291	0.000	0.3752	0.4808
	H2O2	0.0460	0.02291	0.080	-0.0068	0.0988

Based on observed means.
The error term is Mean Square(Error) = 0.001.
*. The mean difference is significant at the 0.05 level.

Multiple Comparisons for Bullet Chilli Variety
Dependent Variable:
LSD

(I) Treatment		Mean Difference (I-J)	Std. Error	Sig.	95% Confidence Interval Lower Bound	95% Confidence Interval Upper Bound
AgNO3	Control	.4270*	0.02475	0.000	0.3699	0.4841
	H2O2	.5630*	0.02475	0.000	0.5059	0.6201
	Putrescine	.4630*	0.02475	0.000	0.4059	0.5201
Control	AgNO3	-.4270*	0.02475	0.000	-0.4841	-0.3699
	H2O2	.1360*	0.02475	0.001	0.0789	0.1931
	Putrescine	0.0360	0.02475	0.184	-0.0211	0.0931
H2O2	AgNO3	-.5630*	0.02475	0.000	-0.6201	-0.5059
	Control	-.1360*	0.02475	0.001	-0.1931	-0.0789

	O3		07		1	9
	Control	-0.0180	0.00907	0.082	-0.0389	0.0029
	Putrescine	-.0270*	0.00907	0.018	-0.0479	-0.0061
Putrescine	AgNO3	.1730*	0.00907	0.000	0.1521	0.1939
	Control	0.0090	0.00907	0.350	-0.0119	0.0299
	H2O2	.0270*	0.00907	0.018	0.0061	0.0479

Based on observed means.
The error term is Mean Square(Error) = 0.000.
*. The mean difference is significant at the 0.05 level.

Multiple Comparisons for Bullet Chilli Variety
Dependent Variable:
LSD

(I) Treatment		Mean Difference (I-J)	Std. Error	Sig.	95% Confidence Interval Lower Bound	95% Confidence Interval Upper Bound
AgNO3	Control	-.0620*	0.00169	0.000	-0.0659	-0.0581
	H2O2	.0090*	0.00169	0.001	0.0051	0.0129
	Putrescine	.0090*	0.00169	0.001	0.0051	0.0129
Control	AgNO3	.0620*	0.00169	0.000	0.0581	0.0659
	H2O2	.0710*	0.00169	0.000	0.0671	0.0749
	Putrescine	.0710*	0.00169	0.000	0.0671	0.0749
H2O2	AgNO3	-.0090*	0.00169	0.001	-0.0129	-0.0051
	Control	-.0710*	0.00169	0.000	-0.0749	-0.0671

	Putrescine	-.3123*	0.01961	0.000	-0.3576	-0.2671		Putrescine	-.1000*	0.02475	0.004	-0.1571	-0.0429		Putrescine	0.0000	0.00169	1.000	-0.0039	0.0039
Putrescine	AgNO3	.4583*	0.01961	0.000	0.4131	0.5036	Putrescine	AgNO3	-.4630*	0.02475	0.000	-0.5201	-0.4059	Putrescine	AgNO3	-.0090*	0.00169	0.001	-0.0129	-0.0051
	Control	.1033*	0.01961	0.001	0.0581	0.1486		Control	-0.0360	0.02475	0.184	-0.0931	0.0211		Control	-.0710*	0.00169	0.000	-0.0749	-0.0671
	H2O2	.3123*	0.01961	0.000	0.2671	0.3576		H2O2	.1000*	0.02475	0.004	0.0429	0.1571		H2O2	0.0000	0.00169	1.000	-0.0039	0.0039

Based on observed means.
The error term is Mean Square(Error) = .001.
*. The mean difference is significant at the 0.05 level.

Based on observed means.
The error term is Mean Square(Error) = .001.
*. The mean difference is significant at the 0.05 level.

Based on observed means.
The error term is Mean Square(Error) = 4.304E-6.
*. The mean difference is significant at the 0.05 level.

Table S6. Changes of total MDA content.

3 Days

ANOVA

Source of Variation	*SS*	*df*	*MS*	*F*	*P-value*	*F crit*
Variety	2.4E-07	1	2.4E-07	5.028152	0.039458	4.493998
Treatments	5.43E-05	3	1.81E-05	378.997	4.56E-15	3.238872
Interaction	2.7E-07	3	9E-08	1.885557	0.172727	3.238872
Within	7.64E-07	16	4.77E-08			
Total	5.55E-05	23				

5 Days

ANOVA

Source of Variation	*SS*	*df*	*MS*	*F*	*P-value*	*F crit*
Variety	1.26E-05	1	1.26E-05	208.0396	1.37E-10	4.493998
Treatments	1.38E-05	3	4.62E-06	76.10802	1.1E-09	3.238872
Interaction	8.45E-06	3	2.82E-06	46.42342	4.05E-08	3.238872
Within	9.7E-07	16	6.06E-08			
Total	3.59E-05	23				

7 Days

ANOVA

Source of Variation	*SS*	*df*	*MS*	*F*	*P-value*	*F crit*
Variety	5.13E-06	1	5.13E-06	87.71424	6.78E-08	4.493998
Treatments	4.05E-05	3	1.35E-05	230.3807	2.27E-13	3.238872
Interaction	1.12E-05	3	3.73E-06	63.79412	4.04E-09	3.238872
Within	9.36E-07	16	5.85E-08			
Total	5.77E-05	23				

Multiple Comparisons for Tejaswani Chilli Variety (3 Days)

Dependent Variable: LSD

(I) Treatment		Mean Difference (I-J)	Std. Error	Sig.	95% Confidence Interval Lower Bound	95% Confidence Interval Upper Bound
AgNO3	Control	.0020*	0.00017	0.000	0.0016	0.0024
	H2O2	0.0000	0.00017	1.000	-0.0004	0.0004
	Putrescine	-.0020*	0.00017	0.000	-0.0024	-0.0016
Control	AgNO3	-.0020*	0.00017	0.000	-0.0024	-0.0016
	H2O2	-.0020*	0.00017	0.000	-0.0024	-0.0016
	Putres	-.0040*	0.000	0.000	-0.00	-0.00

Multiple Comparisons for Tejaswani Chilli Variety (5 Days)

Dependent Variable: LSD

(I) Treatment		Mean Difference (I-J)	Std. Error	Sig.	95% Confidence Interval Lower Bound	95% Confidence Interval Upper Bound
AgNO3	Control	-.0010*	0.00023	0.002	-0.0015	-0.0005
	H2O2	-.0010*	0.00023	0.002	-0.0015	-0.0005
	Putrescine	0.0000	0.00023	1.000	-0.0005	0.0005
Control	AgNO3	.0010*	0.00023	0.002	0.0005	0.0015
	H2O2	0.0000	0.00023	1.000	-0.0005	0.0005
	Putr	.0010*	0.000	0.002	0.000	0.001

Multiple Comparisons for Tejaswani Chilli Variety (7 Days)

Dependent Variable: LSD

(I) Treatment		Mean Difference (I-J)	Std. Error	Sig.	95% Confidence Interval Lower Bound	95% Confidence Interval Upper Bound
AgNO3	Control	-.0050*	0.00019	0.000	-0.0054	-0.0046
	H2O2	-.0030*	0.00019	0.000	-0.0034	-0.0026
	Putrescine	-.0045*	0.00019	0.000	-0.0049	-0.0041
Control	AgNO3	.0050*	0.00019	0.000	0.0046	0.0054
	H2O2	.0020*	0.00019	0.000	0.0016	0.0024
	Putres	.0005*	0.000	0.028	0.000	0.000

	cine		17		44	36
H2O2	AgN O3	0.0000	0.000 17	1.000	-0.00 04	0.000 4
	Contr ol	.0020*	0.000 17	0.000	0.001 6	0.002 4
	Putres cine	-.0020*	0.000 17	0.000	-0.00 24	-0.00 16
Putrescine	AgN O3	.0020*	0.000 17	0.000	0.001 6	0.002 4
	Contr ol	.0040*	0.000 17	0.000	0.003 6	0.004 4
	H2O2	.0020*	0.000 17	0.000	0.001 6	0.002 4

Based on observed means.
The error term is Mean Square(Error) = 4.500E-8.
*. The mean difference is significant at the 0.05 level.

Multiple Comparisons for Bullet Chilli Variety

Dependent Variable:
LSD

(I) Treatment		Mean Differ ence (I-J)	Std. Erro r	Sig.	95% Confidence Interval	
					Low er Bou nd	Upp er Bou nd
AgNO3	Contr ol	.0023*	0.000 18	0.000	0.001 9	0.002 7
	H2O2	0.0003	0.000 18	0.141	-0.00 01	0.000 7
	Putres cine	-.0022*	0.000 18	0.000	-0.00 26	-0.00 18
Control	AgN O3	-.0023*	0.000 18	0.000	-0.00 27	-0.00 19

	esci ne		23		5	5
H2O2	Ag NO 3	.0010*	0.000 23	0.002	0.000 5	0.001 5
	Con trol	0.0000	0.000 23	1.000	-0.00 05	0.000 5
	Putr esci ne	.0010*	0.000 23	0.002	0.000 5	0.001 5
Putrescine	Ag NO 3	0.0000	0.000 23	1.000	-0.00 05	0.000 5
	Con trol	-.0010*	0.000 23	0.002	-0.00 15	-0.00 05
	H2 O2	-.0010*	0.000 23	0.002	-0.00 15	-0.00 05

Based on observed means.
The error term is Mean Square(Error) = 7.625E-8.
*. The mean difference is significant at the 0.05 level.

Multiple Comparisons for Bullet Chilli Variety

Dependent Variable:
LSD

	(I) Treatment	Mean Differ ence (I-J)	Std. Erro r	Sig.	95% Confidence Interval	
					Low er Bou nd	Upp er Bou nd
AgNO3	Con trol	0.0002	0.000 17	0.282	-0.00 02	0.000 6
	H2 O2	.0012*	0.000 17	0.000	0.000 8	0.001 6
	Putr esci ne	.0032*	0.000 17	0.000	0.002 8	0.003 6
Control	Ag NO 3	-0.000 2	0.000 17	0.282	-0.00 06	0.000 2

	cine		19		1	9
H2O2	AgN O3	.0030*	0.000 19	0.000	0.002 6	0.003 4
	Contr ol	-.0020*	0.000 19	0.000	-0.00 24	-0.00 16
	Putres cine	-.0015*	0.000 19	0.000	-0.00 19	-0.00 11
Putrescine	AgN O3	.0045*	0.000 19	0.000	0.004 1	0.004 9
	Contr ol	-.0005*	0.000 19	0.028	-0.00 09	-0.00 01
	H2O2	.0015*	0.000 19	0.000	0.001 1	0.001 9

Based on observed means.
The error term is Mean Square(Error) = 5.203E-8.
*. The mean difference is significant at the 0.05 level.

Multiple Comparisons for Bullet Chilli Variety

Dependent Variable:
LSD

(I) Treatment		Mean Differ ence (I-J)	Std. Erro r	Sig.	95% Confidence Interval	
					Low er Bou nd	Upp er Bou nd
AgNO3	Contr ol	-.0012*	0.000 21	0.000	-0.00 17	-0.00 07
	H2O2	-.0010*	0.000 21	0.001	-0.00 15	-0.00 05
	Putres cine	-.0020*	0.000 21	0.000	-0.00 25	-0.00 15
Control	AgN O3	.0012*	0.000 21	0.000	0.000 7	0.001 7

	H2O2	-.0020*	0.00018	0.000	-0.0024	-0.0016
	Putrescine	-.0045*	0.00018	0.000	-0.0049	-0.0041
H2O2	AgNO3	-0.0003	0.00018	0.141	-0.0007	0.0001
	Control	.0020*	0.00018	0.000	0.0016	0.0024
	Putrescine	-.0025*	0.00018	0.000	-0.0029	-0.0021
Putrescine	AgNO3	.0022*	0.00018	0.000	0.0018	0.0026
	Control	.0045*	0.00018	0.000	0.0041	0.0049
	H2O2	.0025*	0.00018	0.000	0.0021	0.0029

Based on observed means.
The error term is Mean Square(Error) = 5.046E-8.
*. The mean difference is significant at the 0.05 level.

	H2O2	.0010*	0.00017	0.000	0.0006	0.0014
	Putrescine	.0030*	0.00017	0.000	0.0026	0.0034
H2O2	AgNO3	-.0012*	0.00017	0.000	-0.0016	-0.0008
	Control	-.0010*	0.00017	0.000	-0.0014	-0.0006
	Putrescine	.0020*	0.00017	0.000	0.0016	0.0024
Putrescine	AgNO3	-.0032*	0.00017	0.000	-0.0036	-0.0028
	Control	-.0030*	0.00017	0.000	-0.0034	-0.0026
	H2O2	-.0020*	0.00017	0.000	-0.0024	-0.0016

Based on observed means.
The error term is Mean Square(Error) = 4.502E-8.
*. The mean difference is significant at the 0.05 level.

	H2O2	0.0002	0.00021	0.365	-0.0003	0.0007
	Putrescine	-.0008*	0.00021	0.005	-0.0013	-0.0003
H2O2	AgNO3	.0010*	0.00021	0.001	0.0005	0.0015
	Control	-0.0002	0.00021	0.365	-0.0007	0.0003
	Putrescine	-.0010*	0.00021	0.001	-0.0015	-0.0005
Putrescine	AgNO3	.0020*	0.00021	0.000	0.0015	0.0025
	Control	.0008*	0.00021	0.005	0.0003	0.0013
	H2O2	.0010*	0.00021	0.001	0.0005	0.0015

Based on observed means.
The error term is Mean Square(Error) = 6.502E-8.
*. The mean difference is significant at the 0.05 level.

Table S7. Changes of total phenolic content.

3 Days

ANOVA

Source of Variation	*SS*	*df*	*MS*	*F*	*P-value*	*F crit*
Variety	0.037446	1	0.037446	12.61874	0.002654	4.493998
Treatments	0.586958	3	0.195653	65.93194	3.17E-09	3.238872
Interaction	0.833481	3	0.277827	93.62351	2.32E-10	3.238872
Within	0.04748	16	0.002967			
Total	1.505364	23				

5 Days

ANOVA

Source of Variation	*SS*	*df*	*MS*	*F*	*P-value*	*F crit*
Variety	0.45375	1	0.45375	276.1178	1.63E-11	4.493998
Treatments	0.117296	3	0.039099	23.79237	3.86E-06	3.238872
Interaction	0.064839	3	0.021613	13.15203	0.000138	3.238872
Within	0.026293	16	0.001643			
Total	0.662178	23				

7 Days

ANOVA

Source of Variation	*SS*	*df*	*MS*	*F*	*P-value*	*F crit*
Variety	0.305102	1	0.305102	30.78049	4.41E-05	4.493998
Treatments	0.635331	3	0.211777	21.36535	7.68E-06	3.238872
Interaction	3.049982	3	1.016661	102.5669	1.16E-10	3.238872
Within	0.158595	16	0.009912			
Total	4.149009	23				

Multiple Comparisons for Tejaswani Chilli Variety
Dependent Variable:
LSD

(3 Days)

(I) Treatment		Mean Difference (I-J)	Std. Error	Sig.	95% Confidence Interval Lower Bound	95% Confidence Interval Upper Bound
AgNO3	Control	-0.0350	0.04574	0.466	-0.1405	0.0705
	H2O2	-.2340*	0.04574	0.001	-0.3395	-0.1285
	Putrescine	-.5260*	0.04574	0.000	-0.6315	-0.4205
Control	AgNO3	0.0350	0.04574	0.466	-0.0705	0.1405
	H2O2	-.1990*	0.04574	0.002	-0.3045	-0.0935
	Putrescine	-.4910*	0.04574	0.000	-0.5965	-0.3855
H2O2	AgN	.2340*	0.045	0.001	0.128	0.339

Multiple Comparisons for Tejaswani Chilli Variety
Dependent Variable:
LSD

(5 Days)

(I) Treatment		Mean Difference (I-J)	Std. Error	Sig.	95% Confidence Interval Lower Bound	95% Confidence Interval Upper Bound
AgNO3	Control	-.2410*	0.03827	0.000	-0.3292	-0.1528
	H2O2	-.1790*	0.03827	0.002	-0.2672	-0.0908
	Putrescine	-0.0230	0.03827	0.564	-0.1112	0.0652
Control	AgNO3	.2410*	0.03827	0.000	0.1528	0.3292
	H2O2	0.0620	0.03827	0.144	-0.0262	0.1502
	Putrescine	.2180*	0.03827	0.000	0.1298	0.3062
H2O2	AgN	.1790*	0.038	0.002	0.090	0.267

Multiple Comparisons for Tejaswani Chilli Variety
Dependent Variable:
LSD

(7 Days)

(I) Treatment		Mean Difference (I-J)	Std. Error	Sig.	95% Confidence Interval Lower Bound	95% Confidence Interval Upper Bound
AgNO3	Control	.7940*	0.08524	0.000	0.5974	0.9906
	H2O2	.6190*	0.08524	0.000	0.4224	0.8156
	Putrescine	0.1280	0.08524	0.172	-0.0686	0.3246
Control	AgNO3	-.7940*	0.08524	0.000	-0.9906	-0.5974
	H2O2	-0.1750	0.08524	0.074	-0.3716	0.0216
	Putrescine	-.6660*	0.08524	0.000	-0.8626	-0.4694
H2O2	AgN	-.6190*	0.085	0.000	-0.81	-0.42

	O3		74		5	5
	Control	.1990*	0.04574	0.002	0.0935	0.3045
	Putrescine	-.2920*	0.04574	0.000	-0.3975	-0.1865
Putrescine	AgNO3	.5260*	0.04574	0.000	0.4205	0.6315
	Control	.4910*	0.04574	0.000	0.3855	0.5965
	H2O2	.2920*	0.04574	0.000	0.1865	0.3975

Based on observed means.
The error term is Mean Square(Error) =0.003.
*. The mean difference is significant at the 0.05 level.

Multiple Comparisons for Bullet Chilli Variety

Dependent Variable:
LSD

(I) Treatment		Mean Difference (I-J)	Std. Error	Sig.	95% Confidence Interval Lower Bound	Upper Bound
AgNO3	Control	-.2380*	0.04318	0.001	-0.3376	-0.1384
	H2O2	-.6270*	0.04318	0.000	-0.7266	-0.5274
	Putrescine	0.0740	0.04318	0.125	-0.0256	0.1736
Control	AgNO3	.2380*	0.04318	0.001	0.1384	0.3376
	H2O2	-.3890*	0.04318	0.000	-0.4886	-0.2894
	Putrescine	.3120*	0.04318	0.000	0.2124	0.4116
H2O2	AgNO3	.6270*	0.04318	0.000	0.5274	0.7266
	Control	.3890*	0.04318	0.000	0.2894	0.4886

	O3		27		8	2
	Control	-0.0620	0.03827	0.144	-0.1502	0.0262
	Putrescine	.1560*	0.03827	0.004	0.0678	0.2442
Putrescine	AgNO3	0.0230	0.03827	0.564	-0.0652	0.1112
	Control	-.2180*	0.03827	0.000	-0.3062	-0.1298
	H2O2	-.1560*	0.03827	0.004	-0.2442	-0.0678

Based on observed means.
The error term is Mean Square(Error) =0.002.
*. The mean difference is significant at the 0.05 level.

Multiple Comparisons for Bullet Chilli Variety

Dependent Variable:
LSD

(I) Treatment		Mean Difference (I-J)	Std. Error	Sig.	95% Confidence Interval Lower Bound	Upper Bound
AgNO3	Control	0.0510	0.02696	0.095	-0.0112	0.1132
	H2O2	-0.0300	0.02696	0.298	-0.0922	0.0322
	Putrescine	.1520*	0.02696	0.000	0.0898	0.2142
Control	AgNO3	-0.0510	0.02696	0.095	-0.1132	0.0112
	H2O2	-.0810*	0.02696	0.017	-0.1432	-0.0188
	Putrescine	.1010*	0.02696	0.006	0.0388	0.1632
H2O2	AgNO3	0.0300	0.02696	0.298	-0.0322	0.0922
	Control	.0810*	0.02696	0.017	0.0188	0.1432

	O3		24		56	24
	Control	0.1750	0.08524	0.074	-0.0216	0.3716
	Putrescine	-.4910*	0.08524	0.000	-0.6876	-0.2944
Putrescine	AgNO3	-0.1280	0.08524	0.172	-0.3246	0.0686
	Control	.6660*	0.08524	0.000	0.4694	0.8626
	H2O2	.4910*	0.08524	0.000	0.2944	0.6876

Based on observed means.
The error term is Mean Square(Error) = 0.011.
*. The mean difference is significant at the 0.05 level.

Multiple Comparisons for Bullet Chilli Variety

Dependent Variable:
LSD

(I) Treatment		Mean Difference (I-J)	Std. Error	Sig.	95% Confidence Interval Lower Bound	Upper Bound
AgNO3	Control	-1.1870*	0.07713	0.000	-1.3649	-1.0091
	H2O2	-.2370*	0.07713	0.015	-0.4149	-0.0591
	Putrescine	-.5370*	0.07713	0.000	-0.7149	-0.3591
Control	AgNO3	1.1870*	0.07713	0.000	1.0091	1.3649
	H2O2	.9500*	0.07713	0.000	0.7721	1.1279
	Putrescine	.6500*	0.07713	0.000	0.4721	0.8279
H2O2	AgNO3	.2370*	0.07713	0.015	0.0591	0.4149
	Control	-.9500*	0.07713	0.000	-1.1279	-0.7721

	Putrescine	.7010*	0.04318	0.000	0.6014	0.8006
Putrescine	AgNO3	-0.0740	0.04318	0.125	-0.1736	0.0256
	Control	-.3120*	0.04318	0.000	-0.4116	-0.2124
	H2O2	-.7010*	0.04318	0.000	-0.8006	-0.6014

Based on observed means.
The error term is Mean Square(Error) =0.003.
*. The mean difference is significant at the 0.05 level.

	Putrescine	.1820*	0.02696	0.000	0.1198	0.2442
Putrescine	AgNO3	-.1520*	0.02696	0.000	-0.2142	-0.0898
	Control	-.1010*	0.02696	0.006	-0.1632	-0.0388
	H2O2	-.1820*	0.02696	0.000	-0.2442	-0.1198

Based on observed means.
The error term is Mean Square(Error) =0.001.
*. The mean difference is significant at the 0.05 level.

	Putrescine	-.3000*	0.07713	0.005	-0.4779	-0.1221
Putrescine	AgNO3	.5370*	0.07713	0.000	0.3591	0.7149
	Control	-.6500*	0.07713	0.000	-0.8279	-0.4721
	H2O2	.3000*	0.07713	0.005	0.1221	0.4779

Based on observed means.
The error term is Mean Square(Error) =0.009.
*. The mean difference is significant at the 0.05 level.

Table S8. Changes of total Flavonoid Content.

3 Days

ANOVA

Source of Variation	*SS*	*df*	*MS*	*F*	*P-value*	*F crit*
Variety	3.298934	1	3.298934	951.1196	1.11E-15	4.493998
Treatments	0.115905	3	0.038635	11.13891	0.00034	3.238872
Interaction	0.391976	3	0.130659	37.67032	1.76E-07	3.238872
Within	0.055496	16	0.003468			
Total	3.86231	23				

5 Days

ANOVA

Source of Variation	*SS*	*df*	*MS*	*F*	*P-value*	*F crit*
Variety	1.403382	1	1.403382	746.3654	7.46E-15	4.493998
Treatments	0.350153	3	0.116718	62.07435	4.94E-09	3.238872
Interaction	0.129559	3	0.043186	22.96801	4.84E-06	3.238872
Within	0.030085	16	0.00188			
Total	1.913179	23				

7 Days

ANOVA

Source of Variation	*SS*	*df*	*MS*	*F*	*P-value*	*F crit*
Variety	0.008214	1	0.008214	6.323861	0.022982	4.493998
Treatments	0.701271	3	0.233757	179.9667	1.55E-12	3.238872
Interaction	0.300315	3	0.100105	77.06965	9.98E-10	3.238872
Within	0.020782	16	0.001299			
Total	1.030582	23				

Multiple Comparisons for Tejaswani Chilli Variety (3 Days)

Dependent Variable: LSD

(I) Treatment		Mean Difference (I-J)	Std. Error	Sig.	95% Confidence Interval Lower Bound	95% Confidence Interval Upper Bound
AgNO3	Control	.1050*	0.03092	0.009	0.0337	0.1763
	H2O2	-.3390*	0.03092	0.000	-0.4103	-0.2677
	Putrescine	0.0350	0.03092	0.290	-0.0363	0.1063
Control	AgNO3	-.1050*	0.03092	0.009	-0.1763	-0.0337
	H2O2	-.4440*	0.03092	0.000	-0.5153	-0.3727
	Putrescine	-0.0700	0.03092	0.053	-0.1413	0.0013
H2O2	AgN	.3390*	0.030	0.000	0.267	0.410

Multiple Comparisons for Tejaswani Chilli Variety (5 Days)

Dependent Variable: LSD

(I) Treatment		Mean Difference (I-J)	Std. Error	Sig.	95% Confidence Interval Lower Bound	95% Confidence Interval Upper Bound
AgNO3	Control	-.1360*	0.02315	0.000	-0.1894	-0.0826
	H2O2	0.0430	0.02315	0.100	-0.0104	0.0964
	Putrescine	.0900*	0.02315	0.005	0.0366	0.1434
Control	AgNO3	.1360*	0.02315	0.000	0.0826	0.1894
	H2O2	.1790*	0.02315	0.000	0.1256	0.2324
	Putrescine	.2260*	0.02315	0.000	0.1726	0.2794
H2O2	AgN	-0.043	0.023	0.100	-0.09	0.010

Multiple Comparisons for Tejaswani Chilli Variety (7 Days)

Dependent Variable: LSD

(I) Treatment		Mean Difference (I-J)	Std. Error	Sig.	95% Confidence Interval Lower Bound	95% Confidence Interval Upper Bound
AgNO3	Control	-.1910*	0.02773	0.000	-0.2549	-0.1271
	H2O2	0.0540	0.02773	0.087	-0.0099	0.1179
	Putrescine	-.1130*	0.02773	0.004	-0.1769	-0.0491
Control	AgNO3	.1910*	0.02773	0.000	0.1271	0.2549
	H2O2	.2450*	0.02773	0.000	0.1811	0.3089
	Putrescine	.0780*	0.02773	0.023	0.0141	0.1419
H2O2	AgN	-0.054	0.027	0.087	-0.11	0.009

	O3		92		7	3
	Control	.4440*	0.03092	0.000	0.3727	0.5153
	Putrescine	.3740*	0.03092	0.000	0.3027	0.4453
Putrescine	AgNO3	-0.0350	0.03092	0.290	-0.1063	0.0363
	Control	0.0700	0.03092	0.053	-0.0013	0.1413
	H2O2	-.3740*	0.03092	0.000	-0.4453	-0.3027

Based on observed means.
The error term is Mean Square(Error) = 0.001.
*. The mean difference is significant at the 0.05 level.

Multiple Comparisons for Bullet Chilli Variety

Dependent Variable:
LSD

(I) Treatment		Mean Difference (I-J)	Std. Error	Sig.	95% Confidence Interval	
					Lower Bound	Upper Bound
AgNO3	Control	0.0580	0.06057	0.366	-0.0817	0.1977
	H2O2	.1400*	0.06057	0.050	0.0003	0.2797
	Putrescine	-.1710*	0.06057	0.022	-0.3107	-0.0313
Control	AgNO3	-0.0580	0.06057	0.366	-0.1977	0.0817
	H2O2	0.0820	0.06057	0.213	-0.0577	0.2217
	Putrescine	-.2290*	0.06057	0.005	-0.3687	-0.0893
H2O2	AgNO3	-.1400*	0.06057	0.050	-0.2797	-0.0003
	Control	-0.0820	0.06057	0.213	-0.2217	0.0577

	O3	0	15		64	4
	Control	-.1790*	0.02315	0.000	-0.2324	-0.1256
	Putrescine	0.0470	0.02315	0.077	-0.0064	0.1004
Putrescine	AgNO3	-.0900*	0.02315	0.005	-0.1434	-0.0366
	Control	-.2260*	0.02315	0.000	-0.2794	-0.1726
	H2O2	-0.0470	0.02315	0.077	-0.1004	0.0064

Based on observed means.
The error term is Mean Square(Error) = 0.001.
*. The mean difference is significant at the 0.05 level.

Multiple Comparisons for Bullet Chilli Variety

Dependent Variable:
LSD

(I) Treatment		Mean Difference (I-J)	Std. Error	Sig.	95% Confidence Interval	
					Lower Bound	Upper Bound
AgNO3	Control	-0.0500	0.04440	0.293	-0.1523	0.0524
	H2O2	-0.0809	0.04440	0.106	-0.1833	0.0214
	Putrescine	.3697*	0.04440	0.000	0.2673	0.4721
Control	AgNO3	0.0500	0.04440	0.293	-0.0524	0.1523
	H2O2	-0.0310	0.04440	0.505	-0.1334	0.0714
	Putrescine	.4197*	0.04440	0.000	0.3173	0.5220
H2O2	AgNO3	0.0809	0.04440	0.106	-0.0214	0.1833
	Control	0.0310	0.04440	0.505	-0.0714	0.1334

	O3	0	73		79	9
	Control	-.2450*	0.02773	0.000	-0.3089	-0.1811
	Putrescine	-.1670*	0.02773	0.000	-0.2309	-0.1031
Putrescine	AgNO3	.1130*	0.02773	0.004	0.0491	0.1769
	Control	-.0780*	0.02773	0.023	-0.1419	-0.0141
	H2O2	.1670*	0.02773	0.000	0.1031	0.2309

Based on observed means.
The error term is Mean Square(Error) = 0.001.
*. The mean difference is significant at the 0.05 level.

Multiple Comparisons for Bullet Chilli Variety

Dependent Variable:
LSD

(I) Treatment		Mean Difference (I-J)	Std. Error	Sig.	95% Confidence Interval	
					Lower Bound	Upper Bound
AgNO3	Control	-.7200*	0.03103	0.000	-0.7916	-0.6484
	H2O2	-.2730*	0.03103	0.000	-0.3446	-0.2014
	Putrescine	-.1210*	0.03103	0.005	-0.1926	-0.0494
Control	AgNO3	.7200*	0.03103	0.000	0.6484	0.7916
	H2O2	.4470*	0.03103	0.000	0.3754	0.5186
	Putrescine	.5990*	0.03103	0.000	0.5274	0.6706
H2O2	AgNO3	.2730*	0.03103	0.000	0.2014	0.3446
	Control	-.4470*	0.03103	0.000	-0.5186	-0.3754

Putrescine	Putrescine	-.3110*	0.06057	0.001	-0.4507	-0.1713
	AgNO3	.1710*	0.06057	0.022	0.0313	0.3107
	Control	.2290*	0.06057	0.005	0.0893	0.3687
	H2O2	.3110*	0.06057	0.001	0.1713	0.4507

Based on observed means.
The error term is Mean Square(Error) = 0.006.
*. The mean difference is significant at the 0.05 level.

Putrescine	Putrescine	.4506*	0.04440	0.000	0.3482	0.5530
	AgNO3	-.3697*	0.04440	0.000	-0.4721	-0.2673
	Control	-.4197*	0.04440	0.000	-0.5220	-0.3173
	H2O2	-.4506*	0.04440	0.000	-0.5530	-0.3482

Based on observed means.
The error term is Mean Square(Error) = 0.003.
*. The mean difference is significant at the 0.05 level.

Putrescine	Putrescine	.1520*	0.03103	0.001	0.0804	0.2236
	AgNO3	.1210*	0.03103	0.005	0.0494	0.1926
	Control	-.5990*	0.03103	0.000	-0.6706	-0.5274
	H2O2	-.1520*	0.03103	0.001	-0.2236	-0.0804

Based on observed means.
The error term is Mean Square(Error) = 0.001.
*. The mean difference is significant at the 0.05 level.

Table S9. Assay of Pectin Methylesterase.

3 Days

ANOVA

Source of Variation	*SS*	*df*	*MS*	*F*	*P-value*	*F crit*
Variety	1.815	1	1.815	104.9892	1.96E-08	4.493998
Treatments	3.525	3	1.175	67.96819	2.54E-09	3.238872
Interaction	1.485	3	0.495	28.63341	1.14E-06	3.238872
Within	0.2766	16	0.017288			
Total	7.1016	23				

5 Days

ANOVA

Source of Variation	*SS*	*df*	*MS*	*F*	*P-value*	*F crit*
Variety	6	1	6	310.8808	6.62E-12	4.493998
Treatments	8.46	3	2.82	146.114	7.77E-12	3.238872
Interaction	2.28	3	0.76	39.37824	1.29E-07	3.238872
Within	0.3088	16	0.0193			
Total	17.0488	23				

7 Days

ANOVA

Source of Variation	*SS*	*df*	*MS*	*F*	*P-value*	*F crit*
Variety	0.09375	1	0.09375	6.027728	0.025905	4.493998
Treatments	2.56125	3	0.85375	54.89251	1.21E-08	3.238872
Interaction	4.06125	3	1.35375	87.04039	4.02E-10	3.238872
Within	0.24885	16	0.015553			
Total	6.9651	23				

Multiple Comparisons for Tejaswani Chilli Variety

Dependent Variable: LSD

(I) Treatment		Mean Difference (I-J)	Std. Error	Sig.	95% Confidence Interval Lower Bound	95% Confidence Interval Upper Bound
AgNO3	Control	-1.2000*	0.11867	0.000	-1.4737	-0.9263
	H2O2	0.2000	0.11867	0.130	-0.0737	0.4737
	Putrescine	-.8000*	0.11867	0.000	-1.0737	-0.5263
Control	AgNO3	1.2000*	0.11867	0.000	0.9263	1.4737
	H2O2	1.4000*	0.11867	0.000	1.1263	1.6737
	Putrescine	.4000*	0.11867	0.010	0.1263	0.6737

Multiple Comparisons for Tejaswani Chilli Variety

Dependent Variable: LSD

(I) Treatment		Mean Difference (I-J)	Std. Error	Sig.	95% Confidence Interval Lower Bound	95% Confidence Interval Upper Bound
AgNO3	Control	-.6000*	0.08954	0.000	-0.8065	-0.3935
	H2O2	-1.2000*	0.08954	0.000	-1.4065	-0.9935
	Putrescine	-.4000*	0.08954	0.002	-0.6065	-0.1935
Control	AgNO3	.6000*	0.08954	0.000	0.3935	0.8065
	H2O2	-.6000*	0.08954	0.000	-0.8065	-0.3935
	Putrescine	0.2000	0.08954	0.056	-0.0065	0.4065

Multiple Comparisons for Tejaswani Chilli Variety

Dependent Variable: LSD

(I) Treatment		Mean Difference (I-J)	Std. Error	Sig.	95% Confidence Interval Lower Bound	95% Confidence Interval Upper Bound
AgNO3	Control	0.0000	0.10052	1.000	-0.2318	0.2318
	H2O2	1.0000*	0.10052	0.000	0.7682	1.2318
	Putrescine	1.5000*	0.10052	0.000	1.2682	1.7318
Control	AgNO3	0.0000	0.10052	1.000	-0.2318	0.2318
	H2O2	1.0000*	0.10052	0.000	0.7682	1.2318
	Putrescine	1.5000*	0.10052	0.000	1.2682	1.7318

H2O2	AgNO3	-0.2000	0.11867	0.130	-0.4737	0.0737
	Control	-1.4000*	0.11867	0.000	-1.6737	-1.1263
	Putrescine	-1.0000*	0.11867	0.000	-1.2737	-0.7263
Putrescine	AgNO3	.8000*	0.11867	0.000	0.5263	1.0737
	Control	-.4000*	0.11867	0.010	-0.6737	-0.1263
	H2O2	1.0000*	0.11867	0.000	0.7263	1.2737

Based on observed means.
The error term is Mean Square(Error) =0.021.
*. The mean difference is significant at the 0.05 level.

Multiple Comparisons for Bullet Chilli Variety
Dependent Variable:
LSD

(I) Treatment		Mean Difference (I-J)	Std. Error	Sig.	95% Confidence Interval Lower Bound	Upper Bound
AgNO3	Control	0.0000	0.09469	1.000	-0.2184	0.2184
	H2O2	0.2000	0.09469	0.068	-0.0184	0.4184
	Putrescine	-.6000*	0.09469	0.000	-0.8184	-0.3816
Control	AgNO3	0.0000	0.09469	1.000	-0.2184	0.2184
	H2O2	0.2000	0.09469	0.068	-0.0184	0.4184
	Putrescine	-.6000*	0.09469	0.000	-0.8184	-0.3816
H2O2	AgNO3	-0.2000	0.09469	0.068	-0.4184	0.0184
	Contr	-0.200	0.094	0.068	-0.41	0.018

H2O2	AgNO3	1.2000*	0.08954	0.000	0.9935	1.4065
	Control	.6000*	0.08954	0.000	0.3935	0.8065
	Putrescine	.8000*	0.08954	0.000	0.5935	1.0065
Putrescine	AgNO3	.4000*	0.08954	0.002	0.1935	0.6065
	Control	-0.2000	0.08954	0.056	-0.4065	0.0065
	H2O2	-.8000*	0.08954	0.000	-1.0065	-0.5935

Based on observed means.
The error term is Mean Square(Error) = 0.012.
*. The mean difference is significant at the 0.05 level.

Multiple Comparisons for Bullet Chilli Variety
Dependent Variable:
LSD

(I) Treatment		Mean Difference (I-J)	Std. Error	Sig.	95% Confidence Interval Lower Bound	Upper Bound
AgNO3	Control	.4000*	0.13310	0.017	0.0931	0.7069
	H2O2	-1.8000*	0.13310	0.000	-2.1069	-1.4931
	Putrescine	-.8000*	0.13310	0.000	-1.1069	-0.4931
Control	AgNO3	-.4000*	0.13310	0.017	-0.7069	-0.0931
	H2O2	-2.2000*	0.13310	0.000	-2.5069	-1.8931
	Putrescine	-1.2000*	0.13310	0.000	-1.5069	-0.8931
H2O2	AgNO3	1.8000*	0.13310	0.000	1.4931	2.1069
	Contr	2.2000	0.13	0.000	1.893	2.506

H2O2	AgNO3	-1.0000*	0.10052	0.000	-1.2318	-0.7682
	Control	-1.0000*	0.10052	0.000	-1.2318	-0.7682
	Putrescine	.5000*	0.10052	0.001	0.2682	0.7318
Putrescine	AgNO3	-1.5000*	0.10052	0.000	-1.7318	-1.2682
	Control	-1.5000*	0.10052	0.000	-1.7318	-1.2682
	H2O2	-.5000*	0.10052	0.001	-0.7318	-0.2682

Based on observed means.
The error term is Mean Square(Error) = 0.015.
*. The mean difference is significant at the 0.05 level.

Multiple Comparisons for Bullet Chilli Variety
Dependent Variable:
LSD

(I) Treatment		Mean Difference (I-J)	Std. Error	Sig.	95% Confidence Interval Lower Bound	Upper Bound
AgNO3	Control	0.2000	0.10312	0.088	-0.0378	0.4378
	H2O2	.6000*	0.10312	0.000	0.3622	0.8378
	Putrescine	-.4000*	0.10312	0.005	-0.6378	-0.1622
Control	AgNO3	-0.2000	0.10312	0.088	-0.4378	0.0378
	H2O2	.4000*	0.10312	0.005	0.1622	0.6378
	Putrescine	-.6000*	0.10312	0.000	-0.8378	-0.3622
H2O2	AgNO3	-.6000*	0.10312	0.000	-0.8378	-0.3622
	Contr	-.4000*	0.103	0.005	-0.63	-0.16

	ol	0	69		84	4
Putrescine	Putrescine	-.8000*	0.09469	0.000	-1.0184	-0.5816
	AgNO3	.6000*	0.09469	0.000	0.3816	0.8184
	Control	.6000*	0.09469	0.000	0.3816	0.8184
	H2O2	.8000*	0.09469	0.000	0.5816	1.0184

Based on observed means.
The error term is Mean Square(Error) = 0.013.
*. The mean difference is significant at the 0.05 level.

	ol	*	310		1	9
Putrescine	Putrescine	1.0000*	0.13310	0.000	0.6931	1.3069
	AgNO3	.8000*	0.13310	0.000	0.4931	1.1069
	Control	1.2000*	0.13310	0.000	0.8931	1.5069
	H2O2	-1.0000*	0.13310	0.000	-1.3069	-0.6931

Based on observed means.
The error term is Mean Square(Error) = 0.027.
*. The mean difference is significant at the 0.05 level.

	ol		12		78	22
Putrescine	Putrescine	-1.0000*	0.10312	0.000	-1.2378	-0.7622
	AgNO3	.4000*	0.10312	0.005	0.1622	0.6378
	Control	.6000*	0.10312	0.000	0.3622	0.8378
	H2O2	1.0000*	0.10312	0.000	0.7622	1.2378

Based on observed means.
The error term is Mean Square(Error) = 0.016.
*. The mean difference is significant at the 0.05 level.

Table S10. Silver Content.

7 Days						
**ANOVA**						
Source of Variation	*SS*	*df*	*MS*	*F*	*P-value*	*F crit*
Variety	5.491827	1	5.491827	447.54	2.62E-08	5.317655
Treatments	20.04668	1	20.04668	1633.644	1.55E-10	5.317655
Interaction	3.878307	1	3.878307	316.051	1.03E-07	5.317655
Within	0.098169	8	0.012271			
Total	29.51498	11				
